# Models of Neuronal Stimulus-Response Functions: Elaboration, Estimation, and Evaluation

**DOI:** 10.3389/fnsys.2016.00109

**Published:** 2017-01-12

**Authors:** Arne F. Meyer, Ross S. Williamson, Jennifer F. Linden, Maneesh Sahani

**Affiliations:** ^1^Gatsby Computational Neuroscience Unit, University College LondonLondon, UK; ^2^Eaton-Peabody Laboratories, Massachusetts Eye and Ear InfirmaryBoston, MA, USA; ^3^Department of Otology and Laryngology, Harvard Medical SchoolBoston, MA, USA; ^4^Ear Institute, University College LondonLondon, UK; ^5^Department of Neuroscience, Physiology and Pharmacology, University College LondonLondon, UK

**Keywords:** receptive field, sensory system, neural coding

## Abstract

Rich, dynamic, and dense sensory stimuli are encoded within the nervous system by the time-varying activity of many individual neurons. A fundamental approach to understanding the nature of the encoded representation is to characterize the function that relates the moment-by-moment firing of a neuron to the recent history of a complex sensory input. This review provides a unifying and critical survey of the techniques that have been brought to bear on this effort thus far—ranging from the classical linear receptive field model to modern approaches incorporating normalization and other nonlinearities. We address separately the structure of the models; the criteria and algorithms used to identify the model parameters; and the role of regularizing terms or “priors.” In each case we consider benefits or drawbacks of various proposals, providing examples for when these methods work and when they may fail. Emphasis is placed on key concepts rather than mathematical details, so as to make the discussion accessible to readers from outside the field. Finally, we review ways in which the agreement between an assumed model and the neuron's response may be quantified. Re-implemented and unified code for many of the methods are made freely available.

## Introduction

Sensory perception involves not only extraction of information about the physical world from the responses of various sensory receptors (e.g., photoreceptors in the retina and mechanoreceptors in the cochlea), but also the transformation of this information into neural representations that are useful for cognition and behavior. A fundamental goal of systems neuroscience is to understand the nature of stimulus-response transformations at various stages of sensory processing, and the ways in which the resulting neural representations shape perception.

In principle, the stimulus-response transformation for a neuron or set of neurons could be fully characterized if all possible stimulus input patterns could be presented and neural responses measured for each of these inputs. In practice, however, the space of possible inputs is simply too large to be experimentally accessible. Instead, a common approach is to present a rich and dynamic stimulus that spans a sizeable subset of the possible stimulus space, and then use mathematical tools to estimate a model relating the sensory stimulus to the neural response that it elicits.

Such functional models, describing the relationship between sensory stimulus and neural response, are the focus of this review. Unlike biophysical models that seek to describe the physical mechanisms of sensory processing such as synaptic transmission and channel dynamics, functional models typically do not incorporate details of how the response is generated biologically. Thus, in functional models, the model parameters do not reflect physical properties of the biological system, but are instead abstract descriptors of the stimulus-response transformation. An advantage of this abstraction is that functional models can be versatile and powerful tools for addressing many different questions about neural representation.

Another advantage of the abstract nature of functional models is that the power of recent statistical advances in machine learning can be leveraged to estimate model parameters. In this context it is important to clearly distinguish between models and methods. A model describes the functional form of the stimulus-response function (SRF), i.e., how the stimulus is encoded into a neural response. A method (or algorithm) is then used to find parameters that best describe the given model. Usually, there are a number of different methods that can be used to fit a specific model.

Different methods used for model fitting will involve different specific assumptions. For example, constraints may be placed on the statistical structure that the stimulus must take, or the exact shape of the SRF. Changes in the assumptions can produce different estimates of model parameters, even when the method for fitting remains the same. Therefore, it is crucial to employ techniques that can explicitly quantify how well a given model captures neural response properties. Such a quantification serves as a means of determining whether the fitted model is capable of providing an appropriate description of the underlying stimulus-response transformation.

A major goal of this review is to disentangle the existing arsenal of SRF models and estimation methods, and to provide examples that highlight when they work and when they fail. The first part of the review focuses on describing the different SRF models along with various methods that can be used to fit them. The second part of the review then describes techniques that can be used to evaluate the fitted models.

### Statistical preliminaries

Although the subtleties of hypothesis testing (such as the statistics of multiple comparisons) are widely appreciated in the biological sciences, subtleties of model estimation are rarely discussed, even though the corresponding statistical theory is well-developed. Therefore, it will be useful to define some statistical concepts and terms at the outset of this review. Most models explicitly or implicitly define a probability distribution of responses, given a stimulus and some parameters such as a tuning curve, or the weights of a receptive field. By evaluating the probability of the observed data under this distribution, for a known stimulus but varying parameters, we obtain the *likelihood* function over the model parameters. The parameter values which maximize this function, and thus the probability of the observed data, form the *maximum likelihood estimator* (MLE).

The MLE is not the only possible estimator, and we will sometimes discuss more than one way to estimate the parameters of the same model. An estimator is often evaluated in terms of its *bias* (the expected difference between a parameter estimate based on a data set and the parameter value that actually generated those data), its *expected squared error* (bias squared plus variance), and its *consistency* (whether the bias and variance approach 0 when based on increasing amounts of data). However, it is important to realize that bias, variance and consistency are statistical confections. They only have meaning when data actually arise from a model of the form under consideration. Real neural data will *never* be completely and accurately described by abstract models of the type we discuss here; at best we expect the models to provide a decent approximation to the truth. Thus, while consistency and lack of bias are certainly characteristics of a good estimator, these favorable statistical features do not demonstrate “optimality” even within the assumed model form; the estimator may not select the parameters that provide the best model approximation to data generated by a different process.

Practical proof lies elsewhere, in predictive accuracy: how well can the parameters estimated predict a new response that was not used in the estimation process? This is often assessed by cross-validation. A data set is divided into segments; model parameters are estimated leaving out one of the segments; and the predictive quality of the model fit is evaluated on the segment left out. This procedure can be repeated leaving out each segment in turn and the prediction accuracy averaged to yield a more reliable number.

Ultimately, predictive measures such as these (sometimes in more elaborate guises discussed below) are needed to evaluate the quality of both model *and* estimator. Indeed, many pitfalls of interpretation can be avoided by remembering that all models are wrong, and so the only approachable question is: which one is most useful?

## Part 1: elaboration and estimation

### Receptive-field-based stimulus–response function models

A stimulus–response function (SRF) model parametrizes the response of a neural system to a rich input stimulus: usually a random, pseudo-random or natural sensory stimulus sequence presented under controlled conditions. Although many aspects of system response may be modeled—including behavior, metabolic activity, and local field or surface potentials—we focus here on models that target the activity of individual neurons at the level of action potentials (“spikes”), membrane potential or cytoplasmic calcium concentration. Furthermore, we focus on SRF models that include one or more “spatiotemporal” linear filters (Table 1). These filters encode the way in which the neural response integrates elementary inputs, say light at a point in the visual space or power at an acoustic frequency, over time and sensory space. In a sense, then, these filters represent estimates of the receptive field (RF) properties of a neuron, with each filter indicating a “dimension” or “feature” of the stimulus to which it is sensitive.

**Table 1:** **A summary of the models and estimation methods described in the review**.

**Model**	**Estimator**	**Multiple filters**	**References**
Linear-Gaussian	ML (Linear regression)	No	Theunissen et al., 2000
$\hat{r}={\text{k}}^{\text{T}}\text{s},r˜Normal$	Ridge regression	No	Machens et al., 2004
	ARD/ASD	No	Sahani and Linden, 2003a
	ALD	No	Park and Pillow, 2011b
Linear-Nonlinear Poisson	STA	No	Bussgang, 1952; Chichilnisky, 2001
*r* = *f*(**k**^T^**s**), *r* ~ *Poisson*			
	MID	Yes	Sharpee et al., 2004
	STC	Yes	Brenner et al., 2000
*r* = f(**k**^T^**s**), *r* ~ *Poisson*	ML (Poisson GLM)	No	Truccolo et al., 2005
Linear-Nonlinear Bernoulli	ML (Bernoulli GLM)	No	–
*r* = f(**k**^T^**s**), *r* ~ *Bernoulli*			
*r* = *f*(**k**^T^**s**), *r* ~ *Bernoulli*	CbRF	No	Meyer et al., 2014a
General count model	ML	Yes	Williamson et al., 2015
$\hat{r}$ = ∑_*j*_ *f_j_*(**k**^T^**s**),			
*r* ∈ {0, 1, 2, …}			
Gain control model $\hat{r}=f\left(\frac{{\text{k}}_{0}^{\text{T}}\text{s}-u\left(\text{s}\right)}{v\left(\text{s}\right)}\right)$	STC Logistic regression	No No	Schwartz et al., 2002; Rabinowitz et al., 2011
Input nonlinearity model $\hat{r}=f\left({\text{k}}^{\text{T}}\;\sum_{i=1}^{B}\;b_{i}g_{i}\left(s\right)\right)$	ML	No	Ahrens et al., 2008b
Context model $\hat{r}=\sum_{i}k_{i}g\left(s_{i}\right)\;{\text{Context}}_{i}$	ML	Yes	Ahrens et al., 2008a; Williamson et al., 2016
LNLN cascade $\hat{r}=f\left(\sum_{n=1}^{N}W_{n}g_{n}\left({\text{k}}^{\text{T}}\text{s}\right)\right)$	ML	Yes	Butts et al., 2007, 2011; Schinkel-Bielefeld et al., 2012; McFarland et al., 2013
$\hat{r}=f\left(\sum_{c,n,i}w_{c,n}b_{c,i}g_{i}\left({\text{k}}_{c,n}^{\text{T}}\text{s}\right)\right)$	ML	Yes	Lehky et al., 1992; Vintch et al., 2015; Harper et al., 2016
Generalized quadratic model $\hat{r}=f\left({\text{k}}^{{\left(1\right)}^{\text{T}}}\text{s}+{\text{s}}^{\text{T}}{\text{K}}^{\left(2\right)}\text{s}\right)$	Orthogonalized Wiener kernels	Yes	Rieke et al., 1997; Pienkowski and Eggermont, 2010
	Information-theoretic	Yes	Fitzgerald et al., 2011a; Rajan et al., 2013
$\hat{r}=f\left({\text{k}}^{{\left(1\right)}^{\text{T}}}\text{s}+{\text{s}}^{\text{T}}{\text{K}}^{\left(2\right)}\text{s}\right)$	ML	Yes	Rajan et al., 2013
	Maximum expected likelihood	Yes	Park et al., 2013
Time-varying model	Recursive least-squares filtering	No	Stanley, 2002
$\hat{r}=f\left({\text{k}}_{t}^{\text{T}}\text{s}\right)$	ML	No	Brown et al., 2001; Eden et al., 2004
	Adaptive prior	No	Meyer et al., 2014b

*Red colored quantities indicate model parameters that are fixed prior to parameter estimation*.

The choice of stimulus depends on the sensory modality being investigated and the specific question at hand. However, many stimuli can be represented in a common vector-based format, and then very similar, sometimes even identical, models and estimation methods can be applied across modalities to address a variety of questions. Stimulus sequences are usually represented in discretized time, at a rate dictated by the sampling frequency of the stimulus or else re-sampled to match the timescale on which the neural response varies. For simplicity, we assume that the response is measured with the same temporal precision as the stimulus.

**Figure 1:**
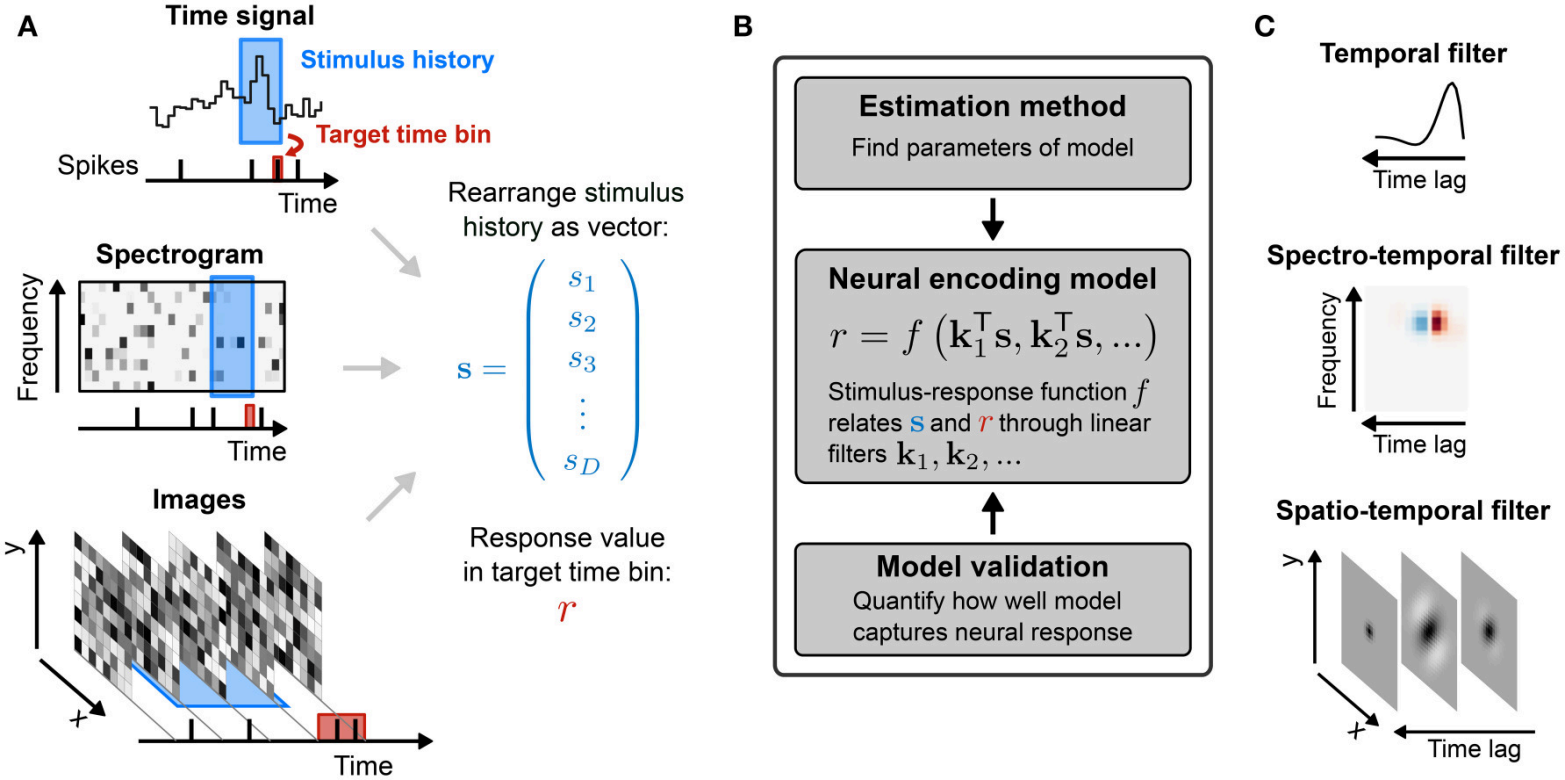
**Sensory stimulus representation for stimulus–response functions**. **(A)** Stimulus examples are sampled from the sensory stimulus representation, e.g., a time signal (top), a spectrogram (middle), or a sequence of image patches (bottom), by rearranging the stimulus history (blue rectangle) as vector *s*. The spike response is usually binned at the temporal resolution of the stimulus, with the target spike bin indicated by the red rectangle. **(B)** The stimulus–response function describes the functional relationship between presented stimulus and measured response. In the models considered here, stimulus and response are related by a linear projection of the stimulus onto one or more linear filters *k*_1_, *k*_2_, …. These filters represent the receptive field of the neuron. **(C)** Once the best parameters for the model have been identified, the representation of the linear filters in the original stimulus space can be interpreted as an an estimate of the stimulus sensitivities of the neuron. Examples of single filters are shown for each type of stimulus representation in **(A)**.

The RF components of an SRF model are most often taken to have limited extent in time (technically, the impulse-response of the filters is finite). Thus, the input used by the model at time *t*, to describe the response *r*(*t*), is limited to a “window” of stimulus values stretching from *t* to some maximal delay τ_max_ time-steps in the past. (The choice of τ_max_ may be guided by biological expectations, or may ultimately be evaluated by measuring the predictive performance of SRF models with differing temporal extents using the methods discussed in the latter part of this review.) The stimulus at each time in this window may be multidimensional, with one value for each pixel in an image, or each frequency band in a spectrogram. It is convenient to collect all such values falling with the window anchored at time *t* into a single (column) vector **s**(*t*) = (*s*_1_(*t*), *s*_2_(*t*), …, *s_D_*(*t*))^T^, with dimension *D* = (length of window) × (dimension of single stimulus frame). Thus, *s*_1_(*t*) might represent the power in the lowest audio frequency channel at time *t*, *s*_64_(*t*) the power in the highest frequency channel also at *t*, *s*_65_(*t*) low-frequency power at *t* − 1 and (say) *s*_640_(*t*) high-frequency power at *t* − 9. The process is illustrated in Figure 1 for different types of stimuli.

**Figure 2:**
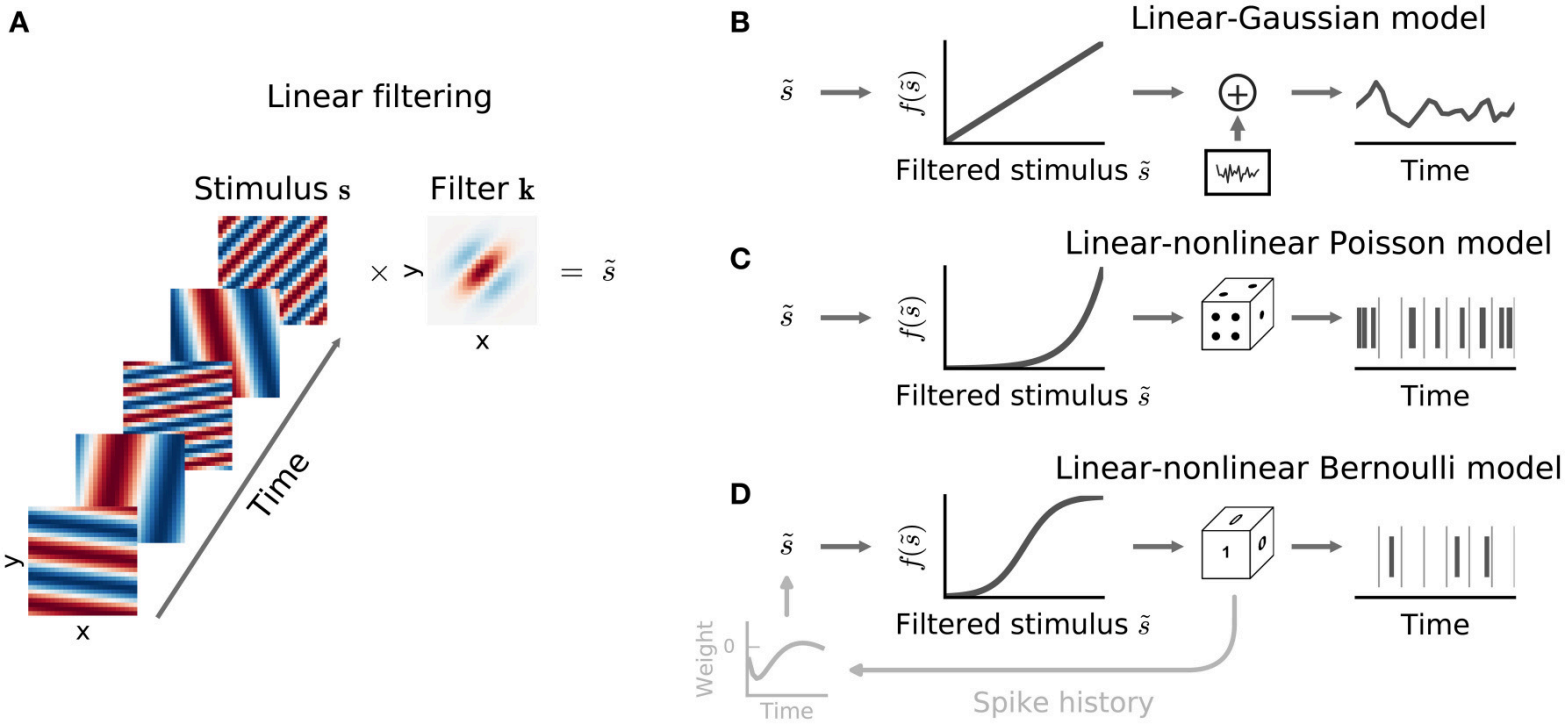
**Common stimulus–response functions**. **(A)** Filtering of stimulus examples through the linear filter **k**. **(B)** (Threshold-)Linear model with Gaussian noise. **(C)** Poisson model with exponential nonlinearity. **(D)** Bernoulli model. All models can be extended using a post-spike filter that indicates dependence of the model's output on the recent response history (light gray).

The discrete-time vector representation of the stimulus allows us to write the action of a single multi-channel linear filter as a inner or “dot” product between the stimulus vector and a vector of filter weights **k** arranged in the same way:

(1) $${\text{k}}^{\text{T}}\text{s}(t)=\sum_{i=1}^{D}k_{i}s_{i}(t)=k_{1}s_{1}(t)+k_{2}s_{2}(t)+\ldots +k_{D}s_{D}(t),$$

thus providing a short-hand notation for integration over space (or channel) as well as over time. The filter **k** is often called a spatio-temporal or spectro-temporal receptive field (STRF) and the weights within it indicate the sensitivity of the neuron to inputs at different points of stimulus space and stimulus history (Figure 2A).

Such discrete-time finite-window vector filtering lies at the heart of the majority of SRF models that have been explored in the literature, although these models may vary in the range of nonlinear transformations that they chain after or before the filtering process to form a “cascade” (Table 1). The cascades range from a simple point-by-point nonlinear transformation that acts on the output of a single linear filter—the linear-nonlinear or LN cascade often employed at earlier sensory stages—to more complicated series or parallel arrangements of filters with multiple intervening nonlinear functions. Some cascades are inspired by a feed-forward description of the sensory pathway, with architectures that recapitulate pathway anatomy. Nonetheless, the assumptions that integration within each stage is linear, often that the nonlinear functions fall within a constrained class, and particularly that responses do not depend on internal state or recurrence, mean that even anatomically inspired SRF models should be regarded as abstract functional models of computation rather than as biologically plausible models of mechanism.

### The linear-gaussian model

In the simplest case the response is assumed to be modeled directly by the output of a single filter, possibly with a constant offset response:

(2) $$r(t)\approx k^{\left(0\right)}+{\text{k}}^{\text{T}}\text{s}(t).$$

The constant offset *k*^(0)^ can be conveniently absorbed into the RF vector **k** by setting an additional dimension in the stimulus vector **s**(*t*) to 1 at all times, so that the offset becomes the coefficient associated with this added dimension. Thus, we will typically omit explicit reference to (and notation of) the offset term.

In practice, most neurons do not respond the same way each time the same stimulus sequence is repeated, and so even if Equation (2) were a correct model of the *mean* response, the actual response measured on one or a finite number of trials will almost surely be different. We reserve the notation *r*(*t*) for the measured response and write $\hat{r}\left(t\right)$ for the SRF model prediction, so that for the linear model $\hat{r}(t)\equiv$ **k**^T^**s**(*t*).

Given a stimulus and a measured response, estimated filter weights $\hat{\text{k}}$ can be obtained by minimizing the squared difference between the model output and the measured data:

(3) $$\hat{\text{k}}=\;\underset{k}{\text{argmin}}\sum_{t}\left||r(t\right)-{\text{k}}^{\text{T}}\text{s}(t)|{|}^{2}={\left({\text{S}}^{\text{T}}\text{S}\right)}^{-1}{\text{S}}^{\text{T}}\text{r},$$

where **S** is the stimulus design matrix formed by collecting the stimulus vectors as rows, **S** = (**s**(1), **s**(2), …, **s**(*T*))^T^ and **r** is a column vector of corresponding measured responses. The right-hand expression in Equation (3) has a long history in neuroscience (Marmarelis and Marmarelis, 1978), and may be interpreted in many ways. It is the solution to a least-squares regression problem, solved by taking the Moore-Penrose pseudoinverse of **S**; it is a discrete time version of the Wiener filter; and, for spike responses, it may be seen as a scaled “correlation-corrected” spike-triggered average (deBoer and Kuyper, 1968; Chichilnisky, 2001). This latter interpretation follows as the matrix product **S**^T^**r** gives the sum of all stimuli that evoked spikes (with stimuli evoking multiple spikes repeated for each spike in the bin); if divided by the total number of spikes this would be the spike-triggered average (STA) stimulus. The term **S**^T^**S** is the stimulus auto-correlation matrix; pre-multiplying by its inverse removes any structure in the STA that might arise from correlations between different stimulus inputs, leaving an estimate of the RF filter. In this way, the estimated model filter corresponds to a descriptive model of the receptive field obtained by “reverse correlation” (deBoer and Kuyper, 1968) or “white noise analysis” (Marmarelis and Marmarelis, 1978).

Thus, the linear SRF model is attractive for its analytic tractability, its computational simplicity (although see the discussion of regularization below) and its interpretability.

If the mean response of the neuron were indeed a linear function of the stimulus, then linear regression would provide an unbiased estimate of the true RF parameters, regardless of the statistical structure of the stimulus ensemble (Paninski, 2003a) and the nature of the neural response variability. More generally, Equation (3) corresponds to the MLE (see Statistical Preliminaries) for a model in which response variability is Gaussian-distributed with constant variance around the filter output (Figure 2B):

(4) $$r(t)=\;{\text{k}}^{\text{T}}\text{s}(t)+\varepsilon (t),\;\varepsilon (t)˜N(0,\sigma^{2}).$$

By itself, this MLE property is of limited value in this case. The assumption of Gaussian response noise is inappropriate for single-trial spike counts, although it may be better motivated when the responses being modeled are trial-averaged mean rates (Theunissen et al., 2000; Linden et al., 2003), subthreshold membrane potentials (Machens et al., 2004), local field potentials (Mineault et al., 2013), or intracranial electrocorticographical recordings (Mesgarani and Chang, 2012); but even then the assumption of constant variance may be violated. Instead, the value of the probabilistic interpretation lies in access to a principled theory of stabilized (or “regularized”) estimation, and to the potential generalization to nonlinear and non-Gaussian modeling assumptions, both of which we discuss below.

### Linear-nonlinear (LN) cascades

Although valuable as a first description, a linear function rarely provides a quantitatively accurate model of neural responses (e.g., Sahani and Linden, 2003b; Machens et al., 2004). Particularly for spiking responses, an attractive extension is to assume that a linear process of stimulus integration within the RF is followed by a separate nonlinear process of response generation. This leads to the linear-nonlinear (or LN) cascade model:

(5) $$\hat{r}(t)=f\left(\;{\text{k}}^{\text{T}}\text{s}(t)\right),$$

where *f* is a static, memoryless nonlinear function. Unlike some more general nonlinear models described later, the input to the nonlinear stage of this LN cascade is of much lower dimension than the stimulus within the RF. Indeed, in Equation (5) it is a single scalar product—although multi-filter versions are discussed below. This reduction in dimensionality allows both the parameters describing the RF filter **k** and any that describe the nonlinearity *f* to be estimated robustly from fewer data than would be required in the more general case.

**Figure 3:**
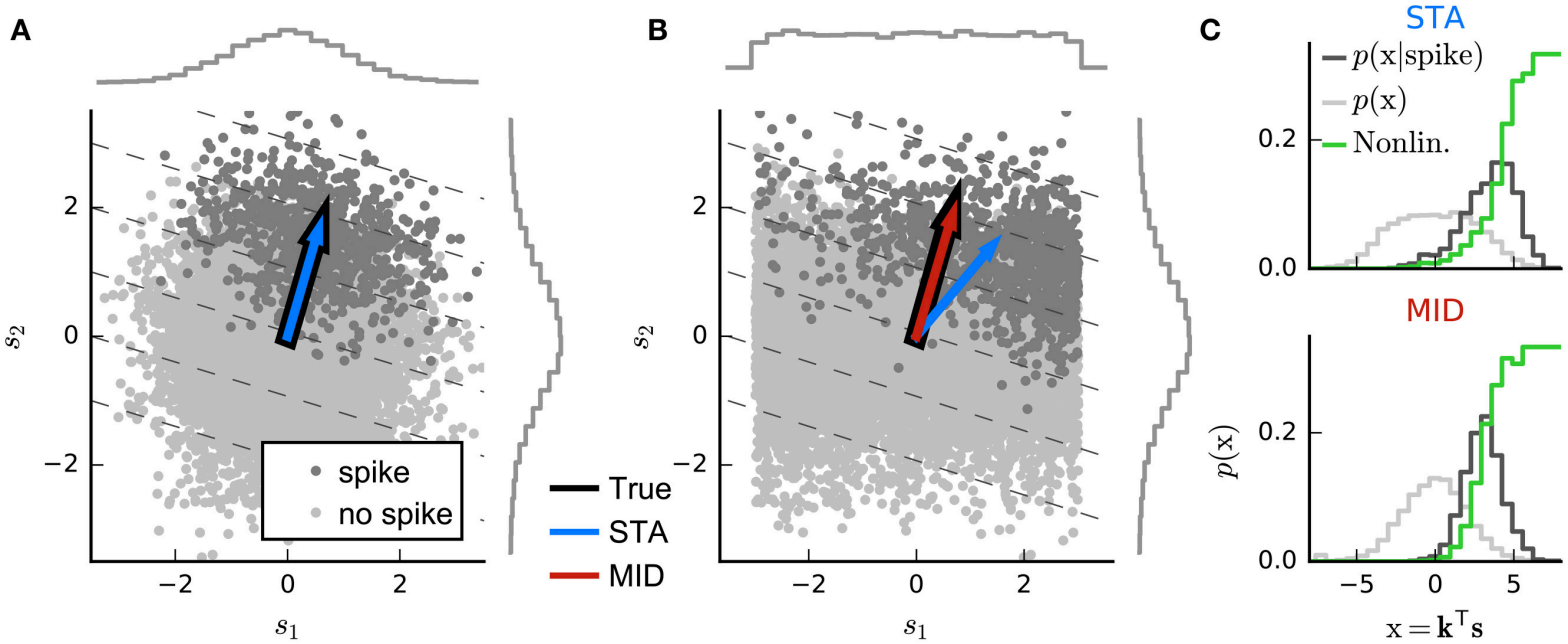
**Geometric illustration of linear filter estimation in the LN model**. **(A)** A two-dimensional stimulus sampled from a Gaussian distribution. Points indicate spike-eliciting (dark gray) and non-spike-eliciting (light gray) stimulus examples with true linear filter shown by the black arrow. For a Gaussian (or more generally, a spherically symmetric) stimulus, the spike-triggered average (STA; blue arrow), given by the mean of all spike-triggered stimuli, recovers the true linear filter. Histograms (insets) show the marginal distributions of stimulus values along each stimulus dimension. Dashed lines indicate “iso-response” hyperplanes (see main text). **(B)** The same as in **(A)** except that stimulus dimension *s*_1_ follows a uniform distribution, resulting in a non-spherically symmetric stimulus distribution. The STA no longer points in the same direction as the true linear filter but the maximally informative dimensions (MID; red arrow) estimator is robust to the change in the stimulus distribution. **(C)** Spike-conditional distribution (*p*(*x*|*spike*)), raw distribution (*p*(*x*)) of filtered stimuli, and histogram-based estimates of the spiking nonlinearity (solid green line) for the STA (top) and MID (bottom) for the example in **(B)**. MID seeks the filter that minimizes the overlap between these distributions. The spiking nonlinearity has been rescaled for visualization.

Indeed, perhaps surprisingly, the linear estimator of Equation (3) may sometimes also provide a useful estimate of the linear-stage RF within an LN model (Bussgang, 1952). To understand when and why, it is useful to visualize the analysis geometrically (Figure 3). Each stimulus vector is represented by a point in a *D*-dimensional space, centered such that origin lies at the mean of the stimulus distribution. Stimuli are colored according to the response they evoke; for spike responses, this distinguishes stimuli associated with action potentials—the “spike-triggered” ensemble—from the “raw” distribution of all stimuli. An RF filter is also a *D*-dimensional vector, and so defines a direction within the space of stimuli. If the neural response can in fact be described by an LN process (with any variability only depending on the stimulus through the value of $\hat{r}\left(t\right)$), then by Equation (5) the stimulus-evoked response will be fully determined by the orthogonal projection of the *D*-dimensional stimulus point onto this RF direction through the dot-product **k**^T^**s**(*t*). Thus, averaging over response variability, the contours defining “iso-response” stimuli will be (hyper)planes perpendicular to the true RF direction.

Now, if the raw stimulus distribution is free of any intrinsic directional bias (that is, it is invariant to rotations about any axis in the *D*-dimensional space, or “spherically symmetric”), the distribution in any such iso-response plane will also be symmetric, so that its mean falls along the RF vector **k**. It follows that the response-weighted mean of all stimuli lies along this same direction, and thus (as long as *f* is not a symmetric function) the empirical response-weighted average stimulus provides an unbiased estimate of the RF. For spike responses, this response-weighted stimulus mean is the STA (Figure 3A). The result can be generalized from spherically symmetric stimulus distributions (Chichilnisky, 2001) to those that can be linearly transformed to spherical symmetry (that is, are elliptically symmetric) (Paninski, 2003a), for which the “correlation-corrected” STA estimator of Equation (3) is consistent.

The symmetry conditions are important to these results. Even small asymmetries may bias estimates away from the true RF as the more heavily sampled regions of the stimulus ensemble are over-weighted in the STA (Figure 3B). With more structured stimulus distributions, including “natural” movies or sounds, the effects of the bias in the STA-based estimators may be profound and misleading. For such stimuli, estimation of an LN model depends critically on assumptions about the functional form of the nonlinearity *f* and the nature of the variability in the response *r*(*t*) (Paninski, 2004; Sharpee et al., 2004).

One intuitive approach is provided by information theory. Consider a candidate RF direction defined by vector $\tilde{k}$, and let $\tilde{s}={\tilde{k}}^{\text{T}}\text{s}$ be the projection of a stimulus point **s** onto this direction. Again making the assumption that the true neural response (and its variability) depends only on the output of an LN process, the predictability of the neural response from $\tilde{s}$ will be maximal and equal to the predictability from the full stimulus vector **s** if and only if $\tilde{k}$ is parallel to the true RF. This predictability can be captured by the mutual information between $\tilde{s}$ and the response, leading to the maximally informative dimensions (MID) estimation approach (Sharpee et al., 2004): identify the direction $\tilde{k}$ for which the empirical estimate of the mutual information between $\tilde{s}\left(t\right)$ and the measured responses *r*(*t*) is maximal.

While this basic statement is independent of assumptions about the nonlinearity or variability, the challenges of estimating mutual information from empirical distributions (Paninski, 2003b) mean that MID-based approaches invariably embody such assumptions in their practical implementations.

### Linear-nonlinear-poisson (LNP) models

For spike-train responses, a natural first assumption is that spike times are influenced only by the stimulus, and are otherwise entirely independent of one another. This assumption requires that the distribution of spike times be governed by a Poisson (point) *process* conditioned on the stimulus, defined by an instantaneous rate function λ(*t*). In turn, this means that the distributions of counts within response time bins of size Δ must follow a Poisson *distribution* (Figure 2C):

(6) $$P(r(t)|\text{s}(t))=\frac{1}{r(t)!}e^{-\lambda (t)\Delta }{\left(\lambda (t)\Delta \right)}^{r(t)};\;\lambda (t)=f({\text{k}}^{\text{T}}\text{s}(t)).$$

The most widely used definition of the MID is based on this assumption of spike-time independence. Again, letting $\tilde{k}$ be a candidate RF direction, and $\tilde{s}$ the value of the projected stimulus, Sharpee et al. (2004) showed that the mutual information between the projected stimuli and independent (and so Poisson-distributed) spikes can be written as a Kullback-Leibler divergence *D*_*KL*_ between the spike-triggered distribution of projected stimuli, $p\left(\tilde{s}|\text{spike}\right)$ and the raw distribution $p\left(\tilde{s}\right)$:

(7) $$I(\tilde{\text{k}})=D_{KL}\left[p(\tilde{s}|\text{spike})\text{ || }p(\tilde{s})\right]=\int p(\tilde{s}|\text{spike})\log\frac{p(\tilde{s}|\text{spike})}{p(\tilde{s})}\text{d}\tilde{s}.$$

The spike-triggered and raw distributions must themselves be estimated to evaluate *I*($\tilde{k}$) and so to identify the MID. The common choice is to estimate each distribution by constructing a binned histogram; and so, in effect, the MID is defined to be the direction along which the histogram of the projected spike-triggered ensemble differs most from the raw stimulus histogram (Figures 3B,C).

Despite the information-theoretic derivation, the Poisson-based information definition combined with histogram-based probability estimates makes the conventional MID approach mathematically identical to a likelihood-based method. Specifically, the histogram-based MID estimate equals the MLE of an LNP model in which the nonlinearity *f* is assumed to be piece-wise constant within intervals that correspond to the bins of the MID histograms (Figure 3C) (Williamson et al., 2015). A corollary is that if these assumptions do not hold, then this form of MID may also be biased. In practice, the approach is also complicated by the fragility of histogram-based estimates of information-theoretic quantities, and by the fact that the objective function associated with such a flexible nonlinearity may have many non-global local maxima, making the true optimum difficult to discover.

Alternative approaches, based either on information theory or on likelihood, assume more restrictive forms of the nonlinearity.

For instance, assuming a Gaussian form for the distributions $p\left(\tilde{s}|spike\right)$ and $p\left(\tilde{s}\right)$ in Equation (7) leads to an estimation procedure that combines both the STA and the spike-triggered-stimulus covariance (STC; see Multi-Filter Models) to identify the RF direction. This has been called “information-theoretic spike-triggered average and covariance” (iSTAC) analysis (Pillow and Simoncelli, 2006). Again, there is a link to a maximum likelihood estimate (this time assuming an exponentiated quadratic nonlinearity) although in this case equivalence only holds if the raw spike distribution is indeed Gaussian, and then too only in the limit as the number of stimuli grows to infinity.

If *f* is assumed to be monotonic and fixed (rather than being defined by parameters that must be fit along with the RF) then Equation (6) describes an instance of a generalized linear model (GLM) (Nelder and Wedderburn, 1972), a widely-studied class of regression models. Many common choices of *f* result in a likelihood which is a concave function (Paninski, 2004), guaranteeing the existence of a single optimum that is easily found by standard optimization techniques such as gradient ascent or Newton's method (see Parameter Optimization). The GLM formulation is also easy to extend to non-Poisson processes, by including probabilistic interactions between spikes in different bins that may be often reminiscent of cellular biophysical processes (see Interactions between bins).

### Non-poisson count models

The LNP model assumes that the exact times of individual spikes, whether in the same or different bins, are entirely statistically independent once their stimulus dependence has been taken into account. While simple, this assumption is rarely biologically justified. Many biophysical and physiological processes lead to statistical dependence between spike times on both short and long timescales. These include membrane refractoriness, spike-rate adaptation, biophysical properties that promote bursting or oscillatory firing, and auto-correlated network input that fluctuates independently of the stimulus. Similar observations apply to other response measures—even to behavioral responses which exhibit clear decision-history dependence (Green, 1964; Busse et al., 2011).

#### Bernoulli models

**Figure 4:**
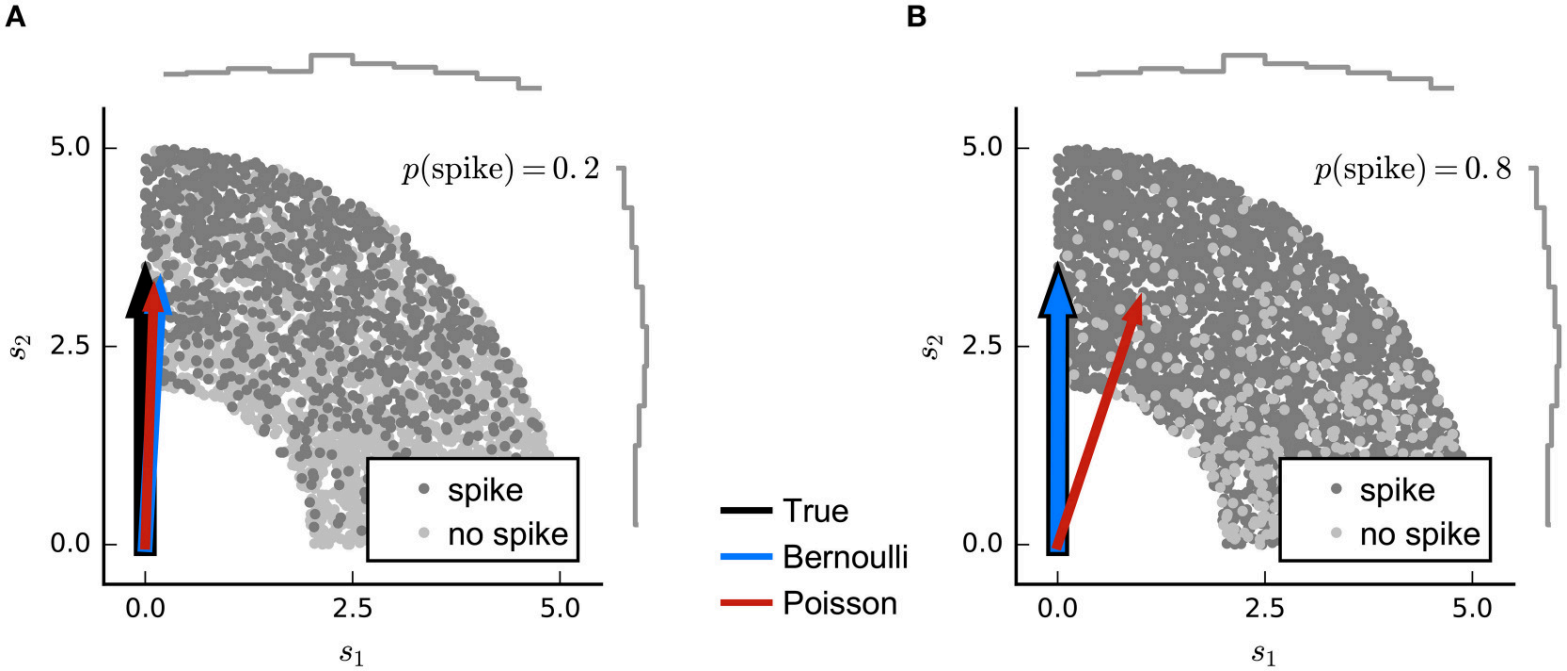
**Simulated example illustrating failure of the Poisson model for Bernoulli distributed responses**. **(A)** *N* = 5000 stimuli were drawn from a uniform distribution on a circular ring. A Bernoulli spike train with *p*(spike) = 0.2 was generated after filtering the 2D stimulus with a RF pointing along the y-axis and a subsequent sigmoid static nonlinear function. Both Poisson GLM (red arrow) and Bernoulli GLM (blue arrow) reliably recover the true filter (black arrow). **(B)** Same as in **(A)** but for *p*(spike) = 0.8. The Poisson GLM estimator fails to recover the true linear filter because its neglects information from silences which are more informative when *p*(no spike) = 1 − *p*(spike) is low (see text). The Bernoulli GLM accounts for silences and thus reliably reconstructs the true linear filter.

The refractoriness of spiking has a strong influence on counts within short time bins. Indeed, when the bin size corresponds to the absolute refractory period (around 1 ms), the observed spike-counts will all be either 0 or 1. If the spike probability is low, the difference between Poisson and binary predictions will be small, and so LNP estimators may still succeed. However, as the probability of spiking in individual bins grows large, an LNP-based estimator (such as MID or the Poisson GLM) may give biased results (Figure 4).

For such short time bins, or for situations in which trial-to-trial variability in spike count is much lower than for a Poisson process (Deweese et al., 2003), a more appropriate LN model will employ a Bernoulli distribution over the two possible responses *r*(*t*) ∈ {0, 1} (Figure 2D):

(8) $$\begin{array}{l} \;\lambda (t)=\frac{1}{\Delta }f({\text{k}}^{\text{T}}\text{s}(t))\\ p(r(t)|\lambda (t))={\left(\lambda (t)\Delta \right)}^{r(t)}{\left(1-\lambda (t)\Delta \right)}^{1-r(t)}, \end{array}$$

where λ(*t*)Δ is now a probability between 0 and 1, and so the maximum possible rate is given by 1/Δ. As for the LNP model, the parameters of this linear-nonlinear-Bernoulli (LNB) model can be estimated using maximum-likelihood methods. The function *f* may be chosen to be piece-wise constant, giving an Bernoulli-based equivalent to the MID approach (Williamson et al., 2015). Alternatively, it may be a fixed, often sigmoid function with values between 0 and 1. In particular, if *f* is the logistic function, the LNB model corresponds to the GLM for logistic regression.

An alternative approach to estimation of the parameters of a binary encoding model is to reinterpret the problem as a classification task in which spike-eliciting and non-spike-eliciting stimuli are to be optimally discriminated (Meyer et al., 2014a). This approach is discriminative rather than probabilistic, and the model can be written as

(9) $$r(t)=\text{H}\left({\text{k}}^{\text{T}}\text{s}(t)-\eta +\varepsilon (t)\right)$$

where η is a spiking threshold and ε(*t*) a random variable reflecting noise around the threshold. *H* is the step function which evaluates to 1 for positive arguments, and 0 otherwise. In this formulation, the RF vector **k** appears as the weight vector of a standard linear classifier; the condition **k**^T^**s**(*t*) = η defines a hyperplane in stimulus space perpendicular to **k** (Figure 3) and stimuli that fall beyond this plane are those that evoke spikes in the model. The noise term creates a probabilistic, rather than hard, transition from 0 to 1 expected spike around the classification boundary. Thus, the optimal weights of this model are determined by minimizing a cost function that depends on the locations of spike-labeled stimuli relative to the associated classification boundary. Robust classifier estimates are often based on objective functions that favor a large *margin*; that is, they set the classification boundary so that the stimuli in the training data that fall nearby and are correctly classified as spike-eliciting or not are assigned as little ambiguity as possible. Such an objective function is the defining characteristic of the support-vector machine (Cortes and Vapnik, 1995). This large-margin approach can be seen as a form of regularization (see the section on Regularization below). Meyer et al. (2014a) report that a large-margin classifier with a fixed objective function gives robust RF estimates for simulated data generated using a wide range of different neural nonlinearities, while a point-process GLM is more sensitive to mismatch between the nonlinearity assumed by the model and that of the data—particularly when working with natural stimuli. On the other hand, logistic regression (i.e., the binary-ouput GLM) also favors large margins when regularized (Rosset et al., 2003) and the results using the classification approach of Meyer et al. (2014a) were very similar to those of the Bernoulli model simulation (Figure 4).

#### Over-dispersed and general count models

Longer bins, for example those chosen to match the refresh rate of a stimulus, may contain more than one spike; but even so the expected distribution of binned counts in response to repeated presentations of the same stimulus will not usually be Poisson.

One form of non-Poisson effect may result from the influence of variability in the internal network state (for instance the “synchronized” and “desynchronized” states of cortical activity; Harris and Thiele, 2011), which may appear to multiplicatively scale the mean of an otherwise Poisson-like response. This additional variance leads to *over-dispersion* relative to the Poisson; that is the Fano factor (variance divided by the mean) exceeds 1. Such over-dispersion within individual bins may be modeled using a “negative binomial” or Polya distribution (Scott and Pillow, 2012). However, the influence of such network effects often extends over many bins or many cells (if recorded together), in which case it may be better modeled as an explicit unobserved variable contributing correlated influence.

More generally, for moderate-length bins where the maximal possible spike count is bounded by refractoriness, the neural response may be described by an arbitrary distribution over the possible count values *j* ∈ {0, …, *r*_max_}. A linear-nonlinear-count (LNC) model can then be defined as:

(10) $$\begin{array}{l} \;\lambda^{\left(j\right)}(t)=f^{\left(j\right)}({\text{k}}^{\text{T}}\text{s}(t))\\ p(r(t)\text{​}=\text{​}j|\lambda^{\left(j\right)}(t))=\lambda^{\left(j\right)}(t) \end{array}$$

with the added constraint on the functions *f*^(*j*)^ that $\overset{r_{\text{max}}}{\underset{j=0}{\sum }}f^{\left(j\right)}\left(x\right)=1$ for all *x*, to ensure that the probabilities over the different counts sum to 1 for each stimulus. This model includes the LNB model as a special case and, as before, the model parameters can be estimated using maximum-likelihood methods. Furthermore, if the functions *f* are assumed to be piece-wise constant, the LNC model estimate of **k** corresponds to a non-Poisson information maximum analogous to the MID. Thus, there is a general and exact equivalence between likelihood-based and information-based estimators for each LN structure (Williamson et al., 2015).

#### Interactions between bins

If responses are measured in short time-bins then longer-term firing interactions such as adaptation, bursting or intrinsic membrane oscillations will induce dependence between counts in different bins. In general, any stimulus-dependent point process can be expressed in a form where the instantaneous probability of spiking depends jointly on the stimulus history and the history of previous spikes, although the spike-history dependence might not always be straightforward. However, a useful approach is to assume a particular parametric form of dependence on past spikes, essentially incorporating these as additional inputs during estimation.

This formulation is perhaps most straightforward within the GLM framework (Chornoboy et al., 1988; Truccolo et al., 2005). For a fixed nonlinearity *f*() we have

(11) $$\lambda (t)=f({\text{k}}^{\text{T}}\text{s}(t)+{\text{g}}^{\text{T}}\text{h}(t))$$

where **g** is a vector of weights and **h**(*t*) is a vector representing the history of spiking at time prior to time *t* (Figure 2); this may be a time-window of response bins stretching some fixed time into the past (as for the stimulus) or may be the outputs of a fixed bank of filters which integrate spike history on progressively longer timescales.

In effect, the combination of **g** and any filters that define **h** serves to implement a “post-spike” filtered input to the intensity function. It is tempting to interpret such filters biophysically as action-potential related influences on the membrane potential of the cell; indeed this model may be seen as a probabilistic version of the spike-response model of Gerstner and Kistler (2002). Suitable forms of post-spike filters may implement phenomena such as refractoriness, bursting or adaptation.

### Multi-filter models

**Figure 5:**
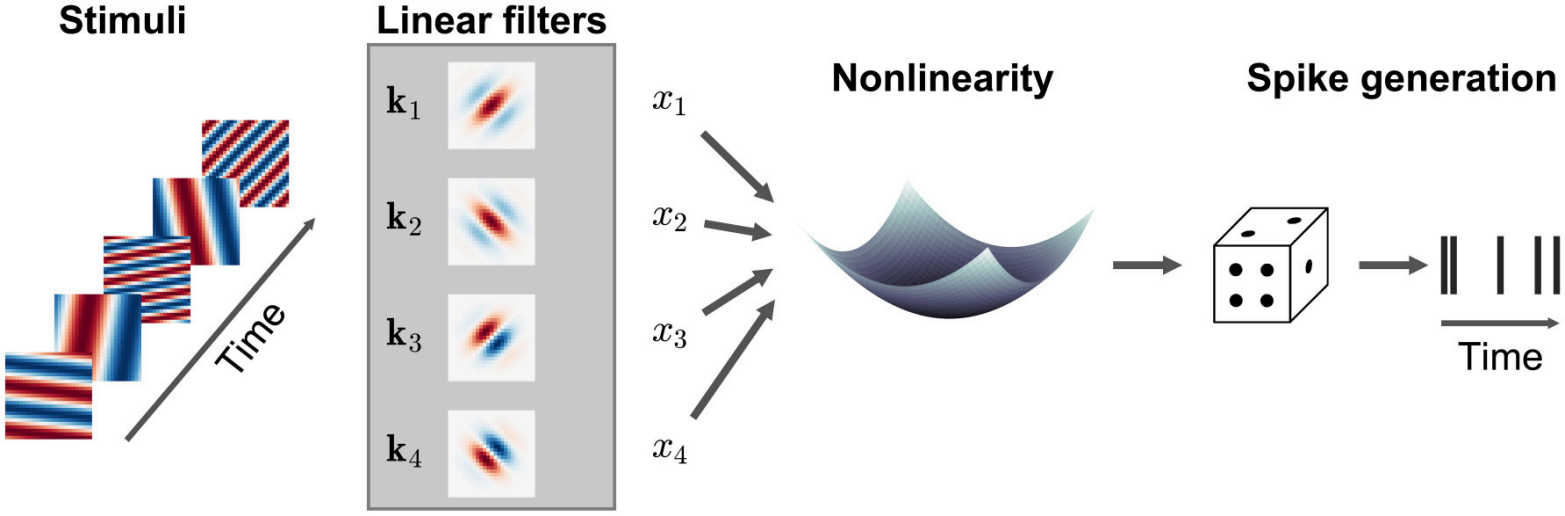
**Illustration of a multi-filter linear-nonlinear Poisson encoding model**. Each input stimulus (here represented by sinusoidal gratings) is filtered by a number of linear filters **k**_1_, **k**_2_, … representing the receptive fields of the neuron. The output of the filters, *x*_1_, *x*_2_, … is transformed by a nonlinearity into an instantaneous spike rate that drives an inhomogeneous Poisson process.

Many LN models can be generalized to incorporate multiple filters acting within the same RF, replacing the single filter **k** by a the matrix **K** = [**k**_1_, **k**_2_, …] where each column represents a different filter (Figure 5). Conceptually, each of these filters may be understood to describe a specific feature to which the neuron is sensitive, although in many cases it is only the subspace of stimuli spanned by the matrix **K** which can be determined by the data, rather than the specific filter shapes themselves. In general, the assumptions embodied in the model or estimators, e.g., regarding the statistical structure of the stimulus, are similar to those made for the single-filter estimation. In particular, the directions in stimulus space (in the sense of Figure 3) along which the spike-triggered covariance (STC) of the stimulus vectors differs from the overall covariance of all stimuli used in the experiment provides one estimate of the columns of **K** in an LNP model (Brenner et al., 2000). This approach to estimation is often called STC analysis. The STC estimate is unbiased provided the overall stimulus distribution is spherically or elliptically symmetric (as was the case for the STA estimator of a single-filter model) *and* the stimulus dimensions are independent or can be linearly transformed to be independent of each other (Paninski, 2003a; Schwartz et al., 2006). These conditions are met only by a Gaussian stimulus distribution, and in other cases the bias can be very significant (Paninski, 2003a; Fitzgerald et al., 2011a).

The MID approach can also be extended to the multi-filter LNP case, defining a subspace projection for a candidate matrix $\tilde{K}$ to be $\tilde{\text{s}}(t)={\tilde{K}}^{\text{T}}\text{s}(t)$ and adjusting $\tilde{K}$ to maximize the Kullback-Leibler divergence between the distributions $p\left(\tilde{\text{s}}|\text{spike}\right)$ and $p\left(\tilde{\text{s}}\right)$. Unfortunately, estimation difficulties make it challenging to use MID to robustly estimate the numbers of filters that might be needed to capture realistic responses (Rust et al., 2005). The problem is not the number of filter parameters *per se* (these scale linearly with stimulus dimensionality), but rather the number of parameters that are necessary to specify the densities $p\left(\tilde{\text{s}}\right)$ and $p\left(\tilde{\text{s}}|\text{spike}\right)$. For common histogram-based density estimators, the number of parameters grows exponentially with dimension (*m* bins for *p* filters requires *m*^*p*^ parameters), e.g., a model with four filters and 25 histogram bins would require fitting 390625 parameters, a clear instance of the “curse of dimensionality.”

In this context, the likelihood-based LN approaches may provide more robust estimates. Rather than depending on estimates of the separate densities, the LN model framework directly estimates a single nonlinear function *f*($\tilde{s}$). This immediately halves the number of parameters needed to characterize the relationship between $\tilde{s}$ and the response. Furthermore, for larger numbers of filters, *f* may be parametrized using sets of basis functions whose numbers grow less rapidly than the number of histogram bins, and which can be tailored to a given data set. This allows estimates of multi-filter LNP models for non-Gaussian stimulus distributions to be extended to a greater number of filters than would be possible with histogram-based MID (Williamson et al., 2015).

In general, multi-filter LN models in which the form of the nonlinearity *f* is fixed have been considered much less widely than in the single-filter case. In part this is because such fixed-*f* models are not GLMs (except in the trivial case where the multiple filter outputs are first summed and then transformed, which is no different to a model with a single filter **k** = ∑_*n*_ **k**_*n*_). Thus, likelihood-based estimation does not benefit from the structural guarantees conferred by the GLM framework. However, there are a few specific forms of nonlinearity which have been considered. One appears in certain models of stimulus-strength gain control, which are considered next. Furthermore, some Input Nonlinearity Models, discussed later, combine multiple filters in more complicated arrangements. Finally, low-rank versions of quadratic, generalized-quadratic and higher-order models (see Quadratic and Higher-Order Models) can also be seen as forms of multi-filter LNP model with fixed nonlinearity.

### Gain control models

Neurons throughout the nervous system exhibit nonlinear behaviors that are not captured by the cascaded models with linear filtering stage or have a more specialized structure than the general multi-filter models described above. For example, the magnitude of the linear filter in a LN model may change with the amplitude (or contrast) of the stimulus (Rabinowitz et al., 2011), or the response may be modulated by stimulus components outside the neuron's excitatory RF (e.g., Chen et al., 2005). These nonlinear behaviors can be attributed to a mechanism known as gain control, in which the neural response is (usually suppressively) modulated by the magnitude of a feature of the stimulus overall. Gain control is a specific form of normalization, a generic principle that is assumed to underlie many computations in the sensory system (for a review see Carandini and Heeger, 2011).

While there are a number of models specific to particular sensory areas and modalities, most gain control models assume the basic form

(12) $$\hat{r}(t)=f\left(\frac{{\text{k}}_{0}^{\text{T}}\text{s}(t)-u(\text{s}(t))}{v(\text{s}(t))}\right)$$

where **k**_0_ is the excitatory RF filter of the neuron, and *u*(**s**) and *v*(**s**) shift and scale the filter output, respectively, depending on the stimulus **s**. As for the LN model, the adjusted filtered stimulus can be related to the response through a static nonlinear function *f*(·).

Schwartz et al. (2002) estimated an excitatory RF filter by the STA **k**_0_ and a set of suppressive filters {**k**_*n*_} by looking for directions in which the STC (built from stimuli orthogonalized with respect to **k**_0_) was smaller than the overall stimulus covariance. They then fit a nonlinearity of the form

(13) $$r(t)=\frac{{\left[{\text{k}}_{0}^{\text{T}}\text{s}(t)\right]}_{+}^{p}}{{\left(\sum_{n}w_{n}|{\text{k}}_{n}^{\text{T}}\text{s}(t){|}^{2}\right)}^{p/2}+\sigma^{2}}$$

finding MLEs for the exponent *p*, which determines the shape of the contrast-response function; the constant σ; and the weights *w*_*n*_, the coefficients with which each of the suppressive filters **k**_*n*_ affect the gain.

While in the above example the excitatory and the suppressive filters acted simultaneously on the stimulus, the gain can also depend on the recent stimulation history. Recent studies demonstrated that a gain control model as in Equation (12) can also account for a rescaling of response gain of auditory cortical neurons depending on the recent stimulus contrast (Rabinowitz et al., 2011, 2012). Specifically, contrast-dependent changes in neural gain could be described by the model

(14) $$r(t)=r_{0}+\frac{c}{1+\exp\left(-\left(\frac{{\text{k}}_{0}^{\text{T}}\text{s}(t)-u\left(\text{s}(t)\right)}{v\left(\text{s}(t)\right)}\right)\right)}$$

where *r*_0_ is the spontaneous rate, *c* a constant, **k**_0_ is the STRF, and *u* and *v* are linear functions of a single “contrast kernel” that characterizes sensitivity to the recent stimulus contrast. In this specific case, the nonlinear function *f* is taken to be the logistic function.

### Input nonlinearity models

LN models assume that any nonlinearity in the neural response can be captured after the output of an initial linear filtering stage. In fact, nonlinear processes are found throughout the sensory pathway, from logarithmic signal compression at the point of sensory transduction, through spiking and circuit-level nonlinearities at intermediate stages, to synaptic and dendritic nonlinearities at the immediate inputs to the cells being studied. *Input* nonlinearities such as these are not captured by a LN model and even the incorporation of a simple static nonlinearity prior to integration (an NL cascade model) can increase the performance of a linear or LN model considerably (Gill et al., 2006; Ahrens et al., 2008a; Willmore et al., 2016).

In the simplest case, the same nonlinear function *g*() may be assumed to apply pointwise to each dimension of **s**. For an input nonlinearity model with a single integration filter, we write: $\hat{r}(t)={\text{k}}^{\text{T}}g(\text{s}(t))$. For *g*() to be estimated, rather than assumed, it must be parametrized—but many parametric choices lead to difficult nonlinear optimizations. Ahrens et al. (2008b) suggest a tractable form, by parametrizing *g*() as a linear combination of *B* fixed basis functions *g*_*i*_, so that $g\left(\right)=\sum_{i=1}^{B}b_{i}g_{i}\left(\right)$. This choice leads to the *multilinear* model

(15) $$\hat{r}(t)=\;{\text{k}}^{\text{T}}\sum_{i\;=\;1}^{B}b_{i}g_{i}(\text{s}(t)),$$

which is linear in each of the parameter vectors **k** and **b** = [*b*_1_, *b*_2_, …, *b_B_*] separately. Least-squares estimates of the parameters can be obtained by alternation: **b** is fixed at an arbitrary initial choice, and a corresponding value for **k** found by ordinary least squares; **k** is then fixed at this value and **b** updated to the corresponding least-squares value; and these alternating updates are continued to convergence. The resulting least-squares estimates at convergence correspond to the MLE for a model assuming constant variance Gaussian noise; however a similar alternating strategy can also be used to find the MLE for a generalized multilinear model with a fixed nonlinearity and Poisson or other point-process stochasticity (Ahrens et al., 2008b). Bayesian regularization (see Regularization) can be incorporated into the estimation process by an approximate method known as variational Bayes (Sahani et al., 2013).

**Figure 6:**
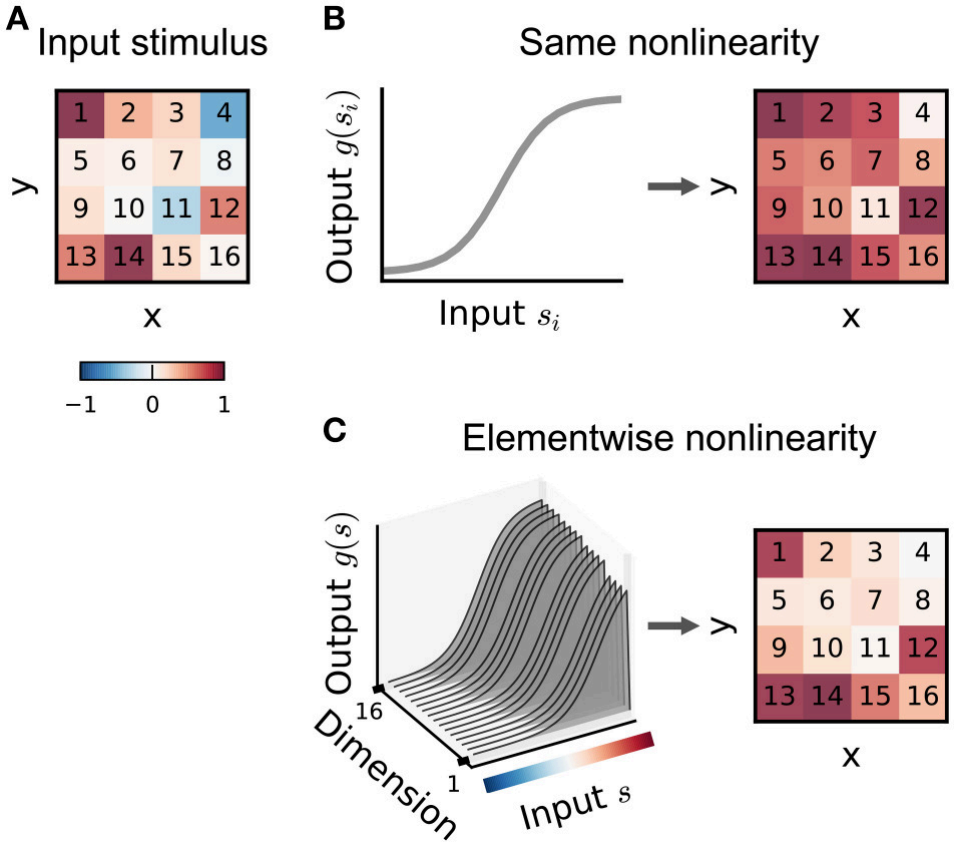
**Illustration of input nonlinearity models**. **(A)** Example image patch stimulus. Numbers indicate dimension indices. **(B)** Input nonlinearity model in which the same nonlinearity (left) acts on all stimulus dimensions, resulting in a transformed stimulus (right). **(C)** Example where the nonlinearity depends on the y dimensions of the stimulus. Colorbar indicates stimulus values in **(A)**.

The multilinear or generalized multilinear formulation may be extended to a broader range of input nonlinearity models. Ahrens et al. (2008a) discuss variants in which different nonlinearities apply at different time-lags or to different input frequency bands in an auditory setting. Indeed, in principle a different combination of basis functions could apply to each dimension of the input (Figure 6), although the number of parameters required in such a model makes it practical only for relatively small stimulus dimensionalities.

**Figure 7:**
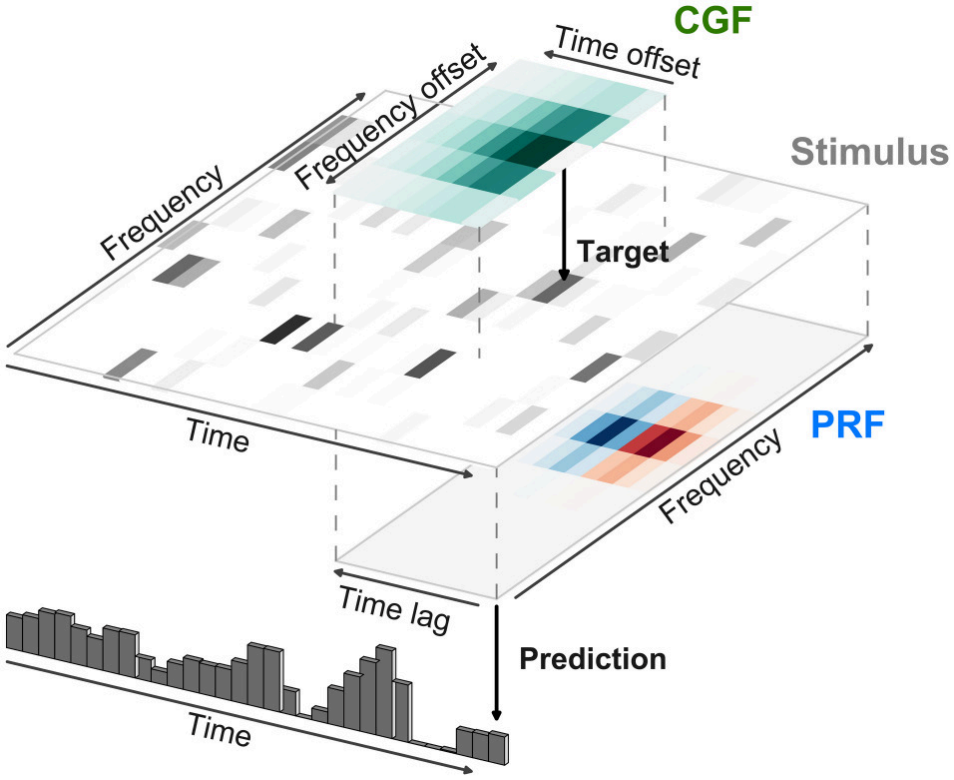
**Modeling of local contextual modulation of the stimulus**. Each element of the input stimulus (here: target tone of an acoustic stimulus) is modulated according to its context using a contextual gain field (CGF). The modulated stimulus is then transformed into a neural response using a principal receptive field (PRF). While each of these stages is linear, the resulting model is nonlinear in the stimulus.

Ahrens et al. (2008a) and Williamson et al. (2016) also introduce multilinear models to capture input nonlinearities in which the sensitivity to each input within the RF is modulated by the local context, for example through multiplicative suppression of repeated inputs (Brosch and Schreiner, 1997; Sutter et al., 1999). The general form of these models is

(16) $$\hat{r}(t)=\sum_{i}k_{i}g(s_{i}(t))\;\cdot \;{\text{Context}}_{i}(t)$$

where the term Context_*i*_(*t*) itself depends on a second local integration field surrounding the *i*^th^ stimulus element (called the contextual gain field or CGF by Williamson et al. 2016). The model as described by Williamson et al. (2016) is illustrated in Figure 7 for an acoustic stimulus. A local window around each input element of the stimulus is weighted by the CGF and integrated to yield a potentially different value of Context_*i*_(*t*) at each element. This value multiplicatively modulates the gain of the response to the element, and the gain-modulated input values are then integrated using weights given by the principal receptive field or PRF. As long as the parameters within Context_*i*_(*t*) appear linearly, the overall model remains multilinear, and can also be estimated by alternating least squares.

Nonlinearities prior to RF integration could also result from more elaborate physiological mechanisms. A simple case might be where an early stage of processing is well described by an LN cascade, and the output from this stage is then integrated at the later stage being modeled. A natural model might then be an LNLN cascade:

(17) $$\hat{r}(t)=f\left(\sum_{n\;=\;1}^{N}w_{n}g_{n}({\text{k}}_{n}^{\text{T}}\text{s}(t))\right)$$

where **k**_*n*_ describes the linear filter and *g*_*n*_ the output nonlinearity of one of the *N* input neurons, and their outputs are combined using weights *w*_*n*_ before a final nonlinear transformation *f*. Such a model has also been called a generalized nonlinear model (GNM) (Butts et al., 2007, 2011; Schinkel-Bielefeld et al., 2012), or nonlinear input model (NIM) (McFarland et al., 2013) and model parameters may be estimated by maximizing the spike-train likelihood of an inhomogeneous Poisson model with rate given by Equation (17)—often using a process of alternation similar to that described above. Vintch et al. (2015) combined an LNLN model with basis-function expansion of the nonlinearity (Equation 15) to yield a model for visual responses of the form

(18) $$\hat{r}(t)=f\left(\sum_{c\;=\;1}^{C}\sum_{n\;=\;1}^{N}w_{c,n}\sum_{i\;=\;1}^{B}b_{c,i}g_{i}({\text{k}}_{c\text{, }n}^{\text{T}}\text{s}(t))\right)$$

in which the parameters *w*_*c,n*_ and *b*_*c,i*_ were fit using alternating least squares, following Ahrens et al. (2008b), with intervening gradient updates of the so-called “subunit” filters **k**_*c,n*_. The subunits were arranged into *C* channels (indexed by *c*). The same nonlinearity applied to all *N* subunits (index *n*) within a channel, and the filters were constrained to be convolutionally arranged; that is, all the **k**_*c,n*_ for each *c* contained the same pattern of spatio-temporal weights shifted to be centered at a different location in space and time. These assumptions helped to contain the potential explosion of parameters, while conforming to biological intuition about the structure of visual processing.

This two-stage convolutional architecture highlights the correspondence between the LNLN structure and a “multilayer perceptron” (MLP) or “artificial neural network” architecture. Indeed, some authors have sought to fit such models with few or no constraints on the filter forms (Lehky et al., 1992; Harper et al., 2016), although such approaches may require substantial volumes of data to provide accurate model estimates.

The methods reviewed thus far in this section have considered models in which the input nonlinearity or a pre-nonlinearity filter are estimated from the neural data. In many cases, however, a fixed input nonlinearity is either assumed from biological intuition, or chosen from amongst a small set of plausible options by explicit comparison of the predictive success of models that assume each in turn (e.g., Gill et al., 2006). For example, models of response functions for central auditory neurons typically assume a stimulus representation based on the modulus (or the square, or logarithm of the modulus) of the short-term Fourier transform of the acoustic stimulus (e.g., the spectrogram illustrated in Figure 1). An alternative approach (reviewed, for the visual system, by Yamins and DiCarlo, 2016) is to base the initial nonlinear transformation on a representation learned from natural stimulus data or a natural task. In particular, DiCarlo and colleagues have exploited the nonlinear internal representations learned within a convolutional neural network trained to classify objects, finding considerable success in predicting responses of the ventral visual pathway based on generalized-linear functions of these representations.

### Quadratic and higher-order models

The cascade nonlinearity models described to this point have been designed to balance biological fidelity and computational tractability in different ways. In principle, it is also possible to characterize nonlinear neural response functions using generic function expansions that are not tailored to any particular expected nonlinear structure.

One approach is to use a polynomial extension of the basic linear model:

(19) $$\begin{array}{l} \hat{r}(t)=k^{\left(0\right)}+\sum_{i\;=\;1}^{D}\;k_{i}^{\left(1\right)}\;s_{i}(t)+\sum_{i,j\;=\;1}^{D}\;k_{ij}^{\left(2\right)}\;s_{i}(t)s_{j}(t)\\ \;+\sum_{i,j,l\;=\;1}^{D}\;k_{ijl}^{\left(3\right)}\;s_{i}(t)s_{j}(t)s_{l}(t)+\ldots , \end{array}$$

where we have re-introduced the explicit constant offset term. Recall that the stimulus vector **s**(*t*) typically includes values drawn from a window in time preceding *t*. This means that the sums range in part over a time index, and so implement (possibly multidimensional) convolutions. Such a convolutional series expansion of a mapping from one time series (the stimulus) to another (the response) is known as a Volterra expansion (Marmarelis and Marmarelis, 1978) and the parameters *k*^(*n*)^ as the Volterra kernels.

While the mapping is clearly nonlinear in the stimulus, Equation (19) is nonetheless linear in the kernel parameters *k*^(*n*)^. Thus, in principle, the MLE of the Volterra expansion truncated at a fixed order *p* could be found by Equation (3), with the parameters concatenated into a single vector: $\overset{ˇ}{\text{k}}=\left[k^{\left(0\right)},k_{1}^{\left(1\right)},k_{2}^{\left(1\right)},\ldots ,k_{D}^{\left(1\right)},k_{11}^{\left(2\right)},k_{12}^{\left(2\right)},\ldots ,k_{DD}^{\left(2\right)},\ldots ,k_{DD\ldots D}^{\left(p\right)}\right]$; and the stimulus vector augmented to incorporate higher-order combinations: $\overset{ˇ}{\text{s}}=\left[1,s_{1},s_{2},\ldots ,s_{D},s_{1}^{2},s_{1}s_{2},\ldots ,s_{D}^{2},\ldots ,s_{D}^{p}\right]$. In practice, this approach raises a number of challenges.

Even if the stimuli used in the experiment are distributed spherically or independently, the ensemble of augmented stimulus vectors $\overset{ˇ}{\text{s}}\left(t\right)$ will have substantial and structured correlation as the higher-order elements depend on the low-order ones. One consequence of this correlation is that the optimal value of any given Volterra kernel depends on the order at which the expansion is truncated; for instance, the linear kernel within the best second-order model will generally differ from the optimal linear fit. If the stimulus distribution is known, then it may be possible to redefine the stimulus terms in Equation (19) (and the entries of $\overset{ˇ}{\text{s}}$) so that each successive order of stimulus entries is made orthogonal to all lower-order values. This re-written expansion is known as a Wiener series, and the corresponding coefficients are the Wiener kernels. The Wiener expansion is best known in the case of Gaussian-distributed stimuli (Rieke et al., 1997), but can also be defined for alternative stimulus classes (Pienkowski and Eggermont, 2010). The orthogonalized kernels can then be estimated in sequence: first the linear, then the quadratic and so on, with the process terminated at the desired maximal order.

However, even if orthogonalized with respect to lower-order stimulus representations, the individual elements of the augmented stimulus at any non-linear order will still be correlated amongst themselves, and so STA (or STC) based analyses will be biased. Thus, estimation depends on explicit least-squares or other maximum-likelihood approaches. This raises a further difficulty, in that computation of the inverse auto-correlation (**S**^T^**S**)^−1^ in Equation (3) may be computationally burdensome and numerically unstable. Park et al. (2013) suggest replacing this term, which depends on the particular stimuli used in the experiment, by its expectation under the distribution used to generate stimuli; for some common distributions, this may be found analytically. This is a maximum expected likelihood (MEL) approach (Ramirez and Paninski, 2014). In a sense, MEL provides an extension of the expected orthogonalization of the Wiener series to structure within a single order of expansion.

The underlying parametric linearity of the Volterra expansion also makes it easy to “generalize” by introducing a fixed, cascaded, output nonlinearity. Although theoretically redundant with the fully general nonlinear expansion already embodied in the Volterra series, this approach provides a simple way to introduce more general nonlinearities when truncating the Volterra expansion at low order. In particular, collecting the second-order Volterra kernel in a matrix ${\text{K}}^{\left(2\right)}=\left[k_{ij}^{\left(2\right)}\right]$ we can write a generalized quadratic model (GQM):

(20) $$\hat{r}(t)=f\left({{\text{k}}^{\left(1\right)}}^{\text{T}}\text{s}(t)+\text{s}{\left(t\right)}^{\text{T}}{\text{k}}^{\left(2\right)}\text{s}(t)\right).$$

Again, as the parameters appear linearly in the exponent, this is a GLM in the (second-order) augmented stimulus **š**, guaranteeing concavity for appropriate choices of *f*() and noise distribution, and rendering the MLE relatively straightforward—although concerns regarding numerical stability remain (Park and Pillow, 2011a). Park et al. (2013) show that MEL can be extended to the GQM for particular combinations of stimulus distribution and nonlinear function *f*. Rajan and Bialek (2013) propose an approach they call “maximally informative stimulus energy” which reduces to MID in **š**. The analysis of Williamson et al. (2015) suggests that this approach would again be equivalent to maximum-likelihood fitting assuming a piece-wise constant nonlinearity and Poisson noise. Finally, the GQM, with logistic nonlinearity and Bernoulli noise, is also equivalent to an information-theoretic approach that seeks to maximize the “noise entropy” of a second-order model of binary spiking (Fitzgerald et al., 2011b).

An obvious further challenge to estimation of truncated Volterra models is the volume of data needed to estimate a number of parameters that grows exponentially in the order *p*. Indeed, this has limited most practical exploration of such expansions to second (i.e., quadratic) order, and often required treatment of stimuli of restricted dimensions (e.g., spectral or temporal, rather than spectro-temporal acoustic patterns, Yu and Young 2000; Pienkowski and Eggermont 2010). One strategy to alleviate this challenge is to redefine the optimization in terms of polynomial “kernel” inner products (a different use of “kernel” from the Volterra parameters) evaluated with respect to each input data point (Sahani, 2000; Franz and Schölkopf, 2006). This approach, often called the “kernel trick,” makes it possible to estimate that part of the higher-order expansion which is determined by the data (a result called the “representer theorem”), and gives access to a powerful theory of optimization and regularization.

A second strategy is to parametrize the higher-order kernels so that they depend on a smaller number of parameters. Many such parametrizations lead to versions of cascade model. Indeed the context-modulated input gain model of Williamson et al. (2016) can be seen as a specific parametrization of the second-order kernel **K**^(2)^. Alternatively, “low-rank” parametrizations of kernels as sums of outer- or tensor-products of vectors lead to versions of LN cascade with polynomial or generalized polynomial nonlinearities. Park et al. (2013) suggest that low-rank quadratic models may be estimated by first estimating the full matrix **K**^(2)^ using MEL, and then selecting the eigenvectors of this matrix corresponding to the largest magnitude eigenvalues. Although consistent, in the sense that the procedure will converge to the generating parameters in artificial data drawn from a low-rank quadratic model, these significant eigenvectors do not generally give the optimal low-rank approximation to real data generated according to some other unknown response function. Instead estimates must be found by direct numerical optimization of the likelihood or expected likelihood. For models of even rank, this optimization may exploit an alternating process similar to that used for multilinear NL formulations (see Williamson et al., 2016, supplementary methods).

A different approach (Theis et al., 2013) extends the parametric spike-triggered framework of Pillow and Simoncelli (2006), using a mixture of Gaussians to model the spike-triggered stimulus distribution and also the distribution of stimuli which did not elicit a spike ($p\left(\tilde{s}|\text{no spike}\right)$; a departure from most spike-triggered estimators). This choice of the spike-absence-triggered distribution rather than $p\left(\tilde{s}\right)$, coupled with a logistic sigmoid nonlinearity and Bernoulli noise, makes this approach similar to a nonlinear version of the classification method described above. The equivalent parametric form is more complex, depending on the log ratio of the two mixture densities; although if the spike-absence-triggered distribution is well modeled as a single Gaussian then this becomes a log-sum of exponentiated quadratic forms.

### Time-varying models

The models described so far seek to characterize neural mechanisms through a combination of linear and nonlinear transformations. These stimulus-response relationships are assumed to be an invariant or stationary property of the neuron, i.e., the linear filters and nonlinearities do not change with time. Whereas this assumption might be reasonable for early sensory areas, neurons at higher stages of sensory processing may have more labile, adaptive and plastic response properties, which fluctuate with changes in stimulus statistics, attentional state, and task demands (e.g., Fritz et al., 2003; Atiani et al., 2009; Rabinowitz et al., 2011; David et al., 2012).

The simplest approach to investigating changes in SRF parameters over time is to split the data into different segments, either sequentially using a moving window (Sharpee et al., 2006) or by (possibly interleaved) experimental condition (Fritz et al., 2003). A separate SRF is then estimated within each segment of the data, with the assumption that the true function remains approximately stationary within it. As the various SRFs are all fit independently, each segment must be sufficiently long to constrain the model parameters, typically requiring recording time on the order of minutes, and thus obscuring more rapid changes. The temporal resolution may be improved to the order of 5–20 s by making the assumption that the fluctuations in SRF parameters are small, and characterizing deviations of the SRF within each segment from a single long-term SRF estimate based on all the data rather than constructing a fully independent estimate for each section. Meyer et al. (2014b) demonstrate this approach, showing that response properties in auditory cortical responses fluctuate at this timescale, and that the resulting non-stationary models therefore describe responses more accurately than stationary models.

To track changes in SRFs at a finer, sub-second, timescales requires that models fit an explicit process describing the evolution of the SRF. Common attempts along these lines, including recursive least-squares filtering (Stanley, 2002) and adaptive point-process estimation (Brown et al., 2001; Eden et al., 2004), can all be described within the framework of state-space models. A state-space model (Chen, 2015) assumes that the temporal variation in model parameters arises through a Markov process; at each time-step, the parameters of the model are determined only by their previous values according to a probabilistic transition process. Such models might include hidden Markov models for discretely labeled states (say, switching between discrete SRF patterns), or linear-Gaussian state space models (related to the Kalman filter) in which parameters evolve continuously. The details of such models are beyond the scope of this review.

### Population interactions

Some changes in the SRFs of individual neurons may be related to population-level changes in the state of the circuit within which they are embedded. For example, transitions between synchronized and desynchronized firing states in cortex are correlated with changes in linear RFs (Wörgötter et al., 1998) and in higher-order stimulus-response properties (Pachitariu et al., 2015). Overall levels of population activity, perhaps associated with similar state transitions, also correlate with multiplicative or additive modulation of tuning curves (Arandia-Romero et al., 2016). Many such population-state changes may be reflected in aggegrate signals such as the local field potential (LFP) (Saleem et al., 2010), and indeed a GLM with a fixed stimulus filter that also incorporated LFP phase information could provide an improved description of neural responses in the anaesthetized auditory cortex (Kayser et al., 2015). Although the parameters of the model are time-invariant, the output of such a model depends on the network dynamics captured by the LFP signal, and thus potentially disentangles intrinsic properties of the neuron from shared network effects.

An alternative approach, in cases where the spiking activity of many neurons has been recorded simultaneously, would be to include in a predictive model for the activity of one neuron, the precise activity of nearby neurons—either directly in a GLM-like structure (Truccolo et al., 2005) or through an inferred latent-space representation of the population such as that found by Gaussian-process Factor Analysis (Yu et al., 2009) and related methods. However, as nearby neurons recorded together may have similar stimulus-response properties, this approach can misattribute stimulus-driven responses to network effects. This is particularly true when the model used is far from correct. Given the option of explaining a stimulus-driven response using an incorrect SRF model, or using a simple (perhaps linear) input from a neighboring neuron with a similar SRF, a population model might find a better fit in the population interaction. Where a sensory stimulus has been presented repeatedly, such model-mismatch effects can be isolated from true trial-by-trial network effects by shuffling neural responses between trials.

### Regularization

**Figure 8:**
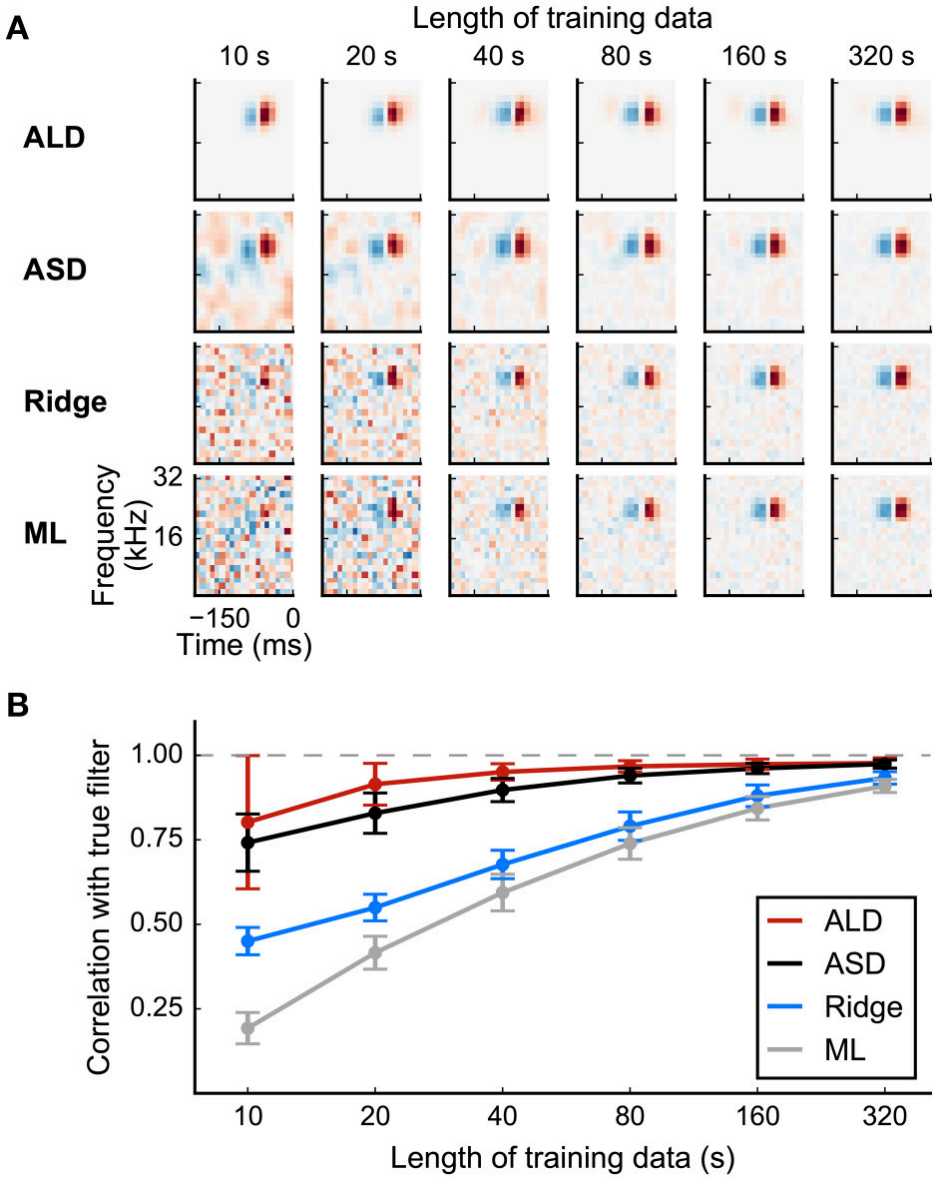
**Simulated example illustrating the effect of priors on linear filter estimation**. Responses were simulated using a linear-Gaussian model with different spectro-temporal receptive fields (STRFs) and a noise-like input stimulus. **(A)** STRF estimates for different data sizes obtained using a linear model with different priors. Maximum-likelihood (ML) estimation and Ridge regression appear noisy for small sample sizes. Estimators using more structured priors like automatic smoothness determination (ASD) and automatic locality determination (ALD) yield better estimates in the low-sampling regime. **(B)** Mean correlation of estimated STRFs with the true STRF across different neurons. Error bars indicate one standard deviation.

Even a linear RF filter may be high-dimensional, possibly containing hundreds or even thousands of elements, particularly when it extends in time as well as over sensory space. An accurate estimate of so many parameters requires a considerable amount of data. In a space of stimuli such as that drawn in Figure 3 the number of dimensions corresponds to the number of RF parameters, and to properly estimate the direction in this space corresponding to the RF, whether by STA, MID, or MLE, each orthogonal axis of this very high-dimensional space must be sampled sufficiently often for the effects of response variability on the estimate of the component of the RF along that axis to average away. However, the difficulty of maintaining stable neural recordings over long times, and other constraints of experimental design, often limit the data available in real experiments. With limited data in very many dimensions, it becomes likely that random variability along some dimensions will happen to fall in a way that appears to be dependent on stimulus value. Simple STA, MID, or MLE estimates cannot distinguish between such random alignment and genuine stimulus-dependence, and so *overfit* to the noise, leading to poor estimates of RF parameters. By construction the overfit model appears to fit data in the training sample as well as possible, but its predictions of responses will fail to generalize to new out-of-sample measurements. The noisy RF estimates might also be biologically implausible, with a “speckled” structure of apparently random sensitivities in time and space (Figure 8).

*Regularized* estimators incorporate strategies to combat overfitting. Two approaches to regularization have seen widespread use in SRF estimation: early stopping and the incorporation of penalty terms in cost functions. In early stopping, the parameters are found by an iterative process, most often gradient ascent in an objective function such as the likelihood or single-spike information. Following each iteration, the predictions of the current parameters are tested on a separate held-out data set (see the Section on Discounting Noise in Evaluation for more on the effect of noise in training and testing data on model fitting). Once these validation predictions no longer improve the iterations are stopped and the current parameters are taken to be the regularized estimate (Sharpee et al., 2004; David et al., 2007; Fitzgerald et al., 2011b).

The second approach to regularization augments the objective function with additional terms or *regularizers* that penalize implausible values of the parameters. In the context of estimation theory, the addition of a regularizer introduces bias into estimates but reduces variance, and so frequently reduces the expected squared error of the estimate. Furthermore, if the magnitude of the regularizer is independent of the number of data, while the scale of the original objective function (such as log-likelihood) grows with the data volume, the regularizer has little impact on the optimum for large data sets, and estimators remain consistent. In practical settings, where the responses do not in fact arise from an instance of the model, regularized estimates are almost always found to generalize more accurately to novel data than unregularized ones.

In the likelihood setting, the regularizer may be interpreted as a *prior* belief about the plausibility of parameter values. Then, by Bayes' rule, the regularized objective corresponds (up to a constant) to the *posterior* belief about the parameters given data, and the maximum of this objective is called the *maximum a posteriori* or MAP estimate:

(21) $${\hat{\text{k}}}_{\text{MAP}}=\text{argmax }[p(\text{r}|\text{S},\text{k})p(\text{k}|\Theta )]$$

(22) =argmax [log p(r|S,k)+log p(k|Θ)]

where *p*(**r**|**S**, **k**) is the probability of the observed response given the stimulus **S** under the model parameters **k** (and so the likelihood function for **k**), and *p*(**k**|Θ) is regularizing prior which may depend on *hyperparameters* Θ (note that taking the logarithm in the second line does not change the location of the maximum). The hyperparameters may be adjusted to refine the penalty term based on the data themselves, either by selecting the values that lead to estimators that generalize best when measured by cross-validation on the training data (Theunissen et al., 2000; Machens et al., 2004; Meyer et al., 2014a), or by a process known variously as *evidence optimization, maximum marginal likelihood*, or sometimes *empirical Bayes* (Sahani and Linden, 2003a). For more complex models such as the multilinear approaches used for NL cascades, the corresponding approach relies on an approximation known as *variational Bayes* (Sahani et al., 2013).

The functional form of the regularizer determines the structure expected in an RF, and conversely the structure that is penalized as implausible. At the most basic level, most regularizers penalize large RF weights. One common choice is a penalty proportional to the sum of squares of weights ($||\text{k}|{|}^{2}=\underset{i}{\sum }k_{i}^{2}$; the “L2 norm”); this is equivalent to assuming a Gaussian prior distribution in the MAP formulation, and is related to a statistical technique called “ridge regression” (Hoerl and Kennard, 1970). This approach strongly penalizes the largest weights, and so tends to favor RF estimates with few extremes. An alternative is to penalize the sum of absolute values ($\left||\text{k}|{|}_{1}=\underset{i}{\sum }|k_{i}\right|$; the “L1 norm”), equivalent to assuming a Laplacian prior and related to “lasso regression” (Tibshirani, 1996). This penalty tends instead to favor sparse RF patterns with a small number of large weights. In both cases, the penalty can be applied after a linear transformation: either ||*R*^T^**k**||^2^ or ||*R*^T^**k**||_1_. If *R* is diagonal, then this approach will simply penalize the weights unequally, favoring larger values in the region with small penalties chosen *a priori*, for instance at small temporal delays. Alternatively, if the columns of *R* evaluate local first or second derivatives in time or space, then such regularizers would favor smooth RF estimates. Smoothness is also promoted by choosing a quadratic form **k**^T^*RR*^T^**k** in which the dominant eigenvectors of matrix (*RR*^T^)^−1^ are themselves smooth patterns.

Evidence optimization methods allow the specific parameters of a regularizer to be adjusted to individual RF estimates rather than set *a priori*. Thus, in *automatic smoothness determination* (ASD; Sahani and Linden, 2003a), the time and length scale of smoothness is optimized for each RF. *Automatic relevance determination* (ARD; also discussed by Sahani and Linden, 2003a) selects the scale of weight penalties enforced by a diagonal *R* automatically, typically resulting in sparse weight distributions with less bias than an L1 approach; and *automatic locality determination* (ALD; Park and Pillow, 2011b) ensures that the large weights are concentrated in a local region of time and space, in concordance with the biological notion of a receptive field. The adaptive methods frequently result in improved model estimates, particularly for small sample sizes. Results are illustrated in Figure 8 for a linear-Gaussian model, comparing maximum-likelihood, “ridge regression,” ASD, and ALD estimates.

### Parameter optimization

Many of the parameter estimators discussed in this review are defined by the optima of likelihood or other objective functions. How easy are these optima to find?

For linear models estimated by least-squares, corresponding to the MLE under the assumption of fixed-variance Gaussian noise, the optimum is available in closed form by Equation (3). This analytic result can be extended to the MAP estimate under the assumption of a fixed zero-mean Gaussian prior with inverse covariance matrix **A** on the RF weights, for which we obtain:

(23) $${\hat{\text{k}}}_{\text{MAP}}={\left({\text{S}}^{\text{T}}\text{S}+\lambda \text{A}\right)}^{-1}{\text{S}}^{\text{T}}\text{r}.$$

The regularization parameter λ is equal to the assumed variance of the Gaussian output noise. In ridge regression, **A** is taken to be the identity matrix, and λ either set arbitrarily or chosen by cross-validation. For adaptive regularizer approaches, including ASD, ARD, and ALD, λ and the matrix **A** must first be found by maximizing the model “evidence” by iterative numerical methods.

With the exception of the STA- and STC-based approaches suited to Gaussian stimulus distributions, estimators for non-linear cascade models require iterative optimization. For a Poisson GLM, possibly with spike-history terms, the log-likelihood function is concave provided that the static nonlinearity assumed is convex and log-concave (Paninski, 2004). This concavity property extends naturally to the log-posterior under a log-concave prior. Such functions have a single unconstrained maximum, which is easily found by gradient-based methods (e.g., Boyd and Vandenberghe, 2004). In particular, a standard algorithm from the GLM literature known as *iteratively reweighted least squares* (IRLS; Green, 1984) exploits information about the expected local curvature of the likelihood to converge rapidly on the optimum. For specific static nonlinearities known as “canonical” (these include the exponential function for Poisson models, and logistic function for Bernoulli models), IRLS corresponds exactly to the Newton method of optimization. In these cases, and if stimuli are drawn randomly from a known and simple distribution, estimation can be further accelerated by maximizing the expected likelihood with only a small cost in accuracy (Ramirez and Paninski, 2014). Alternatively, stochastic gradient techniques estimate gradients using random subsets of the data, converging stably for convex optimization problems (for reviews see Bottou, 1998; Bottou and Bousquet, 2011). These techniques are simple and scalable, making them particularly well-suited to large data sets, and they also facilitate online monitoring of SRF parameters during experiments through their batch-based structure (Meyer et al., 2015).

For estimators based on non-convex objective functions, such as MID, general LN likelihood models, or multilinear NL models, as well as the evidence-optimization stage of some adaptive regularizers, the results of iterative optimization may depend on the parameter value from which the iterations begin. Thus, additional steps are needed to ensure that the local optimum found is likely to represent a good parameter or hyperparameter choice. One approach is to repeat the iterative optimization starting from a number of different initial parameter values, accepting the results of the run that leads to the best value of the objective function (or, as a form of regularization, the best validation performance; compare the discussion of early stopping above). Alternatively, stochastic gradient methods, particularly incorporating momentum terms, may escape poor local extrema and approach the true optimal parameter values (Ruder, 2016). A similar idea, albeit without explicit use of gradient information, underlies stochastic search methods such as simulated annealing (Kirkpatrick et al., 1983). In the general case, however, no approach beyond exhaustive search can guarantee that the value obtained will be the true global optimum of the objective function.

## Part 2: evaluation

Once we have a found the parameters of a model for a set of neural data, there remains the important task of validating the quality of the model fit. In this section, we discuss different methods for quantifying how well a fitted model captures the neural response.

There are different settings in which model performance needs to be evaluated. The relatively straightforward scenario is when we wish to compare the performance of two or more estimators for a specific model, e.g., different regression-based estimators of the linear-Gaussian model (see Equation 2). In this case, the log-likelihood provides a convenient measure for comparing the *relative* performance of the estimators on the same set of validation data. However, often we are interested in finding which model amongst a number of different models provides the best description of the neural response. Again, this is a relative comparison, but in this case of the models rather than the estimators; therefore a *model-independent* measure is required, such as the single-spike information (Brenner et al., 2000; Sharpee et al., 2004).

Ultimately, however, the goal is not only to identify the best of a limited set of models for a recorded set of data, but also to quantify the fraction of the response captured by any of the models. This scenario—evaluation of *absolute* model performance—is more complicated, because response prediction errors arise not only from inaccurate model assumptions but also from variability in neural responses. While these variations might represent an important aspect of the neural response, from a modeling perspective they are usually treated as “noise” (unless the variations are under control of the experimenter or are related to observable variables), and the impact of this “noise” has to be taken into account when evaluating absolute model performance.

In the following, we will provide an overview of common measures used to evaluate performance of the different stimulus-response function models reviewed above. We will also provide an intuitive outline of a method that allows the separation of response prediction errors arising from inaccurate model assumptions from errors arising from noise inherent in neuronal spike trains (Sahani and Linden, 2003b; see also Williamson et al., 2016, supplementary methods).

### Rate-based measures

#### Mean squared error (MSE)

For continuous responses such as spike rates or local field potentials, a natural measure for the quality of an estimated model is the mean squared error (MSE; $\sigma_{e}^{2}$) between the estimated response $\hat{r}$ and the measured response *r*,

(24) $$\sigma_{e}^{2}=\frac{1}{T}\sum_{t=1}^{T}{\left(\hat{r}(t)-r(t)\right)}^{2}$$

(25) $$=\langle {\left(\hat{r}(t)-r(t)\right)}^{2}\rangle ,$$

with 〈·〉 used to denote average over time. The MSE is a common measure of error used in many estimation problems, and is also closely related to the negative log-likelihood of the linear-Gaussian model.

**Figure 9:**
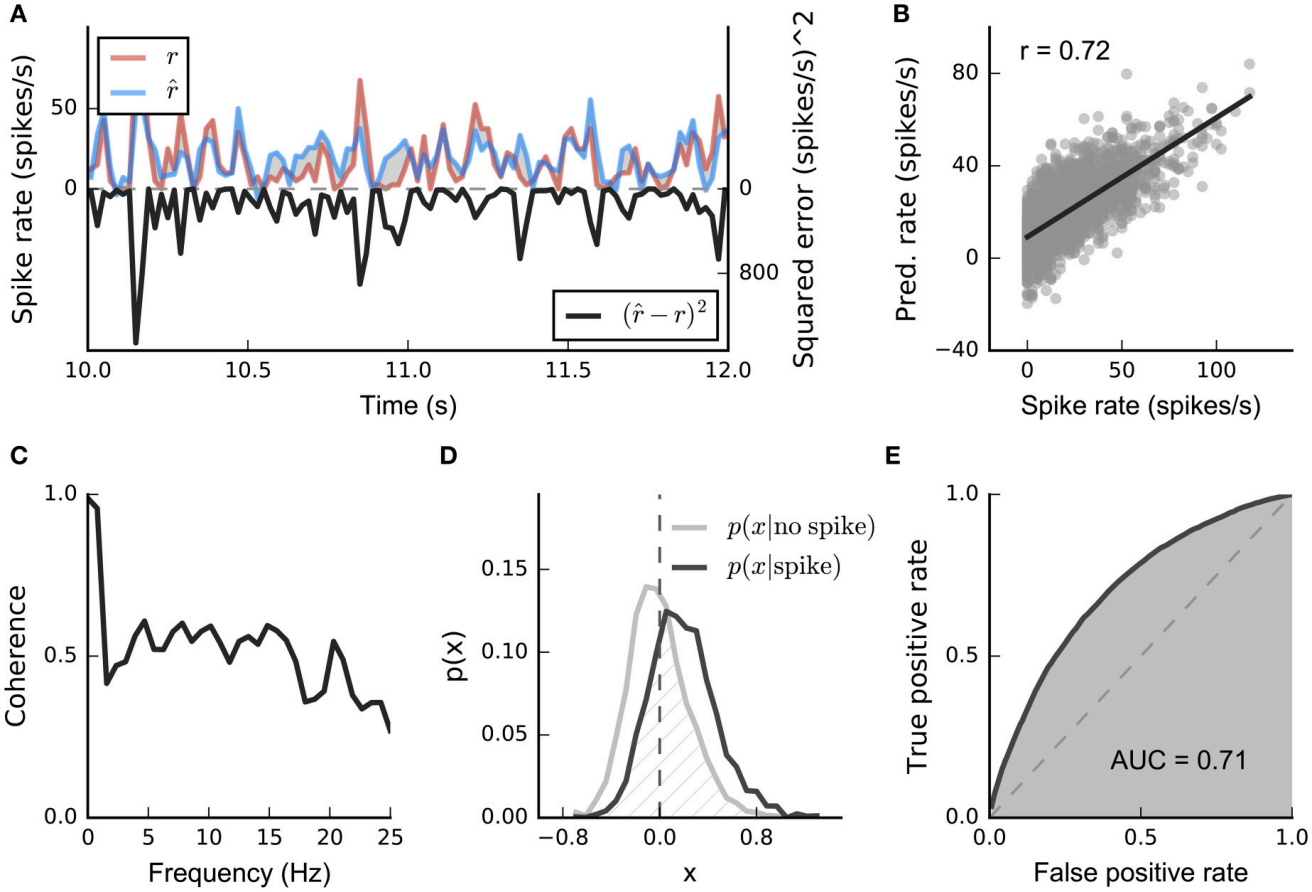
**Common techniques for evaluating SRFs**. **(A)** The mean squared error (MSE) measures the squared error (black line) per time step between the measured rate (red line) and predicted rate (blue line). The error between the rates (gray shaded area) depends on mean and scaling of the rates. **(B)** The correlation coefficient reflects the linearity between measured and predicted response, indicated by the least squares line fit. The correlation is invariant to linear transformations, i.e., its value does not depend on the mean and the scaling of the responses. **(C)** The coherence assesses the linear relationship between two variables in frequency space; i.e., it is a frequency-dependent correlation measure. **(D)** Conditional distribution of filtered stimuli that elicited a spike (black line) or no spike (gray line). The hatched area indicates the overlap between the two distributions, which is related to prediction performance in a binary coding model (see text for details). **(E)** A receiver-operating characteristic curve (ROC) can be constructed from the distributions in **(D)** by computing false positive and true positive rates for all possible thresholds along the *x*-axis. The area under the ROC curve (AUC; shaded gray area) provides a scalar measure of the prediction performance of the fitted model.

The MSE measures the mean error per sample (Figure 9A) but it is not bounded above; higher variability in the recordings will produce higher MSE estimates for equivalent data sizes. This limitation makes it difficult to compare MSE values across different brain areas or even across different recordings from the same area. The coefficient of determination, or *R*^2^ statistic, normalizes the MSE by the variance in the neural response,

(26) $$R^{2}=\frac{\sigma_{r}^{2}-\sigma_{e}^{2}}{\sigma_{r}^{2}}$$

where $\sigma_{r}^{2}=\langle {\left(r\left(t\right)-\langle r\rangle \right)}^{2}\rangle$ is the variance in the neural response about the mean value $\langle r\rangle =1/T\overset{T}{\underset{t=0}{\sum }}r\left(t\right)$ and $\sigma_{e}^{2}$ is the MSE. Unfortunately, *R*^2^ cannot be used directly to quantify how well a model reproduces the recorded response as it does not distinguish stimulus-dependent variance in the response from stimulus-independent variability (“noise”). A modification of Equation (26) described below (see Discounting Noise in Evaluation) makes it possible to measure the fraction of *explainable* variance in the data captured by a specific model in the presence of such stimulus-independent variability.

### Correlation and coherence

Correlation measures the degree of linear dependence between two variables. For a predicted and observed time-varying firing rates, the sample correlation coefficient, also known as the Pearson correlation, is defined as

(27) $$\rho_{r,\hat{r}}=\frac{\text{cov}(r,\hat{r})}{\sigma_{r}\sigma_{\hat{r}}}$$

where $\text{cov}\left(r,\hat{r}\right)=\langle (r\left(t\right)-\langle r\rangle )(\hat{r}\left(t\right)-\langle \hat{r}\rangle )\rangle$ is the sample cross-covariance, $\sigma_{\hat{r}}=\sqrt{\langle {\left(\hat{r}(t)-\langle \hat{r}\rangle \right)}^{2}\rangle }$ is the standard deviation of the model prediction, and σ_*r*_ is that of the measured data. The correlation coefficient is bounded between -1 and 1, with a correlation of 1 indicating a perfect linear relation between predicted and actual response and values close to zero indicating that the responses are linearly unrelated. An example for a cortical neuron is shown in Figure 9B.

The correlation coefficient is centered and normalized and therefore does not depend on mean or scaling of the signals. In settings where the focus is on capturing the temporal modulation of the firing rate rather than its overall magnitude, this may provide an advantage over the MSE. Introducing a time lag between the two signals, and computing a correlation at each lag yields a function known as the crosscorrelogram. This may reveal temporal relationships between the prediction and measurement, such as temporal offsets or correlation lengths, that are not evident from the correlation at zero time lag.

An alternative formulation of the linear dependency between two signals is the magnitude-squared coherence,

(28) $$\gamma^{2}(\omega )=\frac{|S_{r\hat{r}}(\omega ){|}^{2}}{S_{r}(\omega )S_{\hat{r}}(\omega )},$$

where $S_{r\hat{r}}\left(\omega \right)$ is the cross-spectrum of *r* and $\hat{r}$, and *S*_*r*_(ω) and $S_{\hat{r}}\left(\omega \right)$ are the power spectra of *r* and $\hat{r}$, respectively (Gardner, 1992). The coherence measures the strength of the linear relationship between two processes as a function of frequency. While it can be more expensive to compute, it has several important advantages over the time-domain correlation. First, for spike data, the correlation coefficient and correlogram require binned spike counts and their values depend on the bin size. As Fourier transforms of spike-train signals can be found without explicit discretization or smoothing, computation of the coherence does not require binning and is less sensitive to the bin size if the data have been pre-binned. The temporal scale of the correlation is instead implicit in the frequency range over which the coherence is considered. Thus, the coherence may be diagnostically valuable, revealing for instance that a model accurately predicts slow fluctuations in the response while missing many short time-scale events (Figure 9C). For nonstationary signals, such as stimulus-driven firing rates, the coherence must be estimated from continuous time-varying quantities. Common approaches for obtaining continuous firing rate estimates include moving-window averaging, wavelet-based filtering, and multitaper techniques (Brown et al., 2004).

### Spike-based measures

#### Single-spike information

The single-spike information (Equation 7) maximized by the MID estimator of the LNP model provides a measure of the mutual information between stimulus and response, regardless of the shape of the neural nonlinearity. Furthermore, it does not depend on the scaling of the linear filter(s) which might be inherently different for different estimators. Therefore, it is a useful measure to use for comparing different LNP models.

However, empirical estimation of information-theoretic quantities from finite data is non-trivial. Histogram-based estimation of single-spike information values can result in substantial upward bias in information estimates (Brenner et al., 2000; Paninski, 2003b). While it is possible to correct for this bias to some degree, the optimal number of histogram bins also depends on the amount of data (Paninski, 2003b). Thus, the parametrization of the histogram-based estimator must be chosen carefully, or investigated as a variable.

Once an appropriate parametrization has been identified, the single-spike information can be normalized by the total information in the response (Brenner et al., 2000). The total information can be estimated from a large number (e.g., 50–150) of repetitions of a short stimulus segment (Sharpee et al., 2008), using

(29) $$I_{\text{resp}}=\frac{1}{T}\int \text{d}t\frac{r(t)}{\langle r\rangle }\log_{2}\frac{r(t)}{\langle r\rangle },$$

where *r*(*t*) is the time-varying firing rate for the stimulus segment averaged over all stimulus repetitions, and 〈*r*〉 is the overall mean firing rate across time and repetitions. Finite data effects both in the single-spike information and the total information in the response can be reduced by (linear) extrapolation to infinite data (Sharpee et al., 2008).

#### ROC analysis

The problem of correctly predicting a spike can also be phrased in terms of a detection task with the goal of successfully detecting spike-eliciting stimuli from a distribution of stimuli that mostly fail to evoke spikes. In signal detection theory, success in detecting a desired event may be quantified by the receiver operating characteristic (ROC) curve, which is generated by plotting the fraction of correctly detected spike stimuli (“true positive rate”) vs. the fraction of falsely detected non-spike stimuli (“false positive rate”) for different spiking thresholds (Green and Swets, 1966; Meyer et al., 2014a). Because the output of most binary SRF models depends only on the filtered stimulus, this is equivalent to “shifting” the threshold along the axis defined by the filter and estimating the rates from the conditional distributions.

This is illustrated in Figure 9 for an example auditory cortical neuron. The overlap between the distributions can be quantified by integrating over all thresholds (e.g., using the trapezium rule) yielding the area under the ROC curve (AUC). A value close to 1 corresponds to a small overlap, whereas a value close to 0.5 indicates that the spike- and non-spike-stimulus distributions overlap substantially along the RF direction identified by the model.

From a model perspective, the overlap determines the amount of noise in the system. The discriminative approach described above (see Bernoulli Models) seeks to find the filter in stimulus space that minimizes the overlap between the distributions, which is equivalent to finding the model with minimum coding noise (Meyer et al., 2014a). Note that the single-spike information seeks to minimise the overlap between similar conditional distributions in terms of the Kullback-Leibler divergence (see Figure 3D). In case the number of spikes is small relative to the number of bins, which is typically the case for small enough bin sizes, AUC and single-spike information are highly correlated, with AUC exhibiting considerably smaller bias and variance for small sample sizes (Meyer et al., 2013).

### Discounting noise in evaluation

The response of a neuron to repeated presentations of the same physical stimulus can vary considerably, even in anaesthetized preparations (Tolhurst et al., 1983; Goris et al., 2014). This variability makes it difficult both to estimate the parameters of the model in the first place, and then to quantify the extent to which a given model or class of models has captured the true response of the neuron. Here, we describe a three-step procedure for finding the fraction of the explainable component in the response that can be captured by a model, for a population of similar neurons (e.g., from a specific brain area). We also illustrate the principles on simulated data.

#### Signal and noise power

Suppose that we have available the responses of a population of neurons to *N* repetitions of the same stimulus. (It is not essential that all neurons were recorded at once as the analysis is performed treating each neuron as a separate sample.) Our objective is to measure the performance of a predictive model in terms of the fraction of the neuron's response that it successfully predicts. Following Sahani and Linden (2003b) we focus on the *response power* or response variance $\sigma_{r}^{2}$ (see Equation 26).

From a modeling perspective, the response to the *n*th stimulus repetition, *r*^(*n*)^(*t*), may be divided into a reliable (stimulus-driven *signal*) part μ(*t*) and a variable (*noise*) component η^(*n*)^(*t*),

(30) $$r^{\left(n\right)}(t)=\mu (t)+\eta^{\left(n\right)}(t).$$

We define μ(*t*) to be the expected response to the stimulus—the average that would be obtained from an infinite collection of responses to the same stimulus—and so η^(*n*)^(*t*) has an expected value of zero for all *t* and *n*. The signal μ(*t*) reflects the time-locked, stimulus-driven part of the response of the neuron under consideration, and it is thus the component of the response that is (in theory) predictable by a model of the cell's SRF. However, the average of a finite number of trial responses collected within experimental constraints will retain a contribution from the noise, and thus the true signal response cannot be determined. Nevertheless, it is possible to form an unbiased estimator of the *power* or variance in that response $\sigma_{\mu }^{2}=\langle {\left(\mu \left(t\right)-\langle \mu \rangle \right)}^{2}\rangle$ as follows.

**Figure 10:**
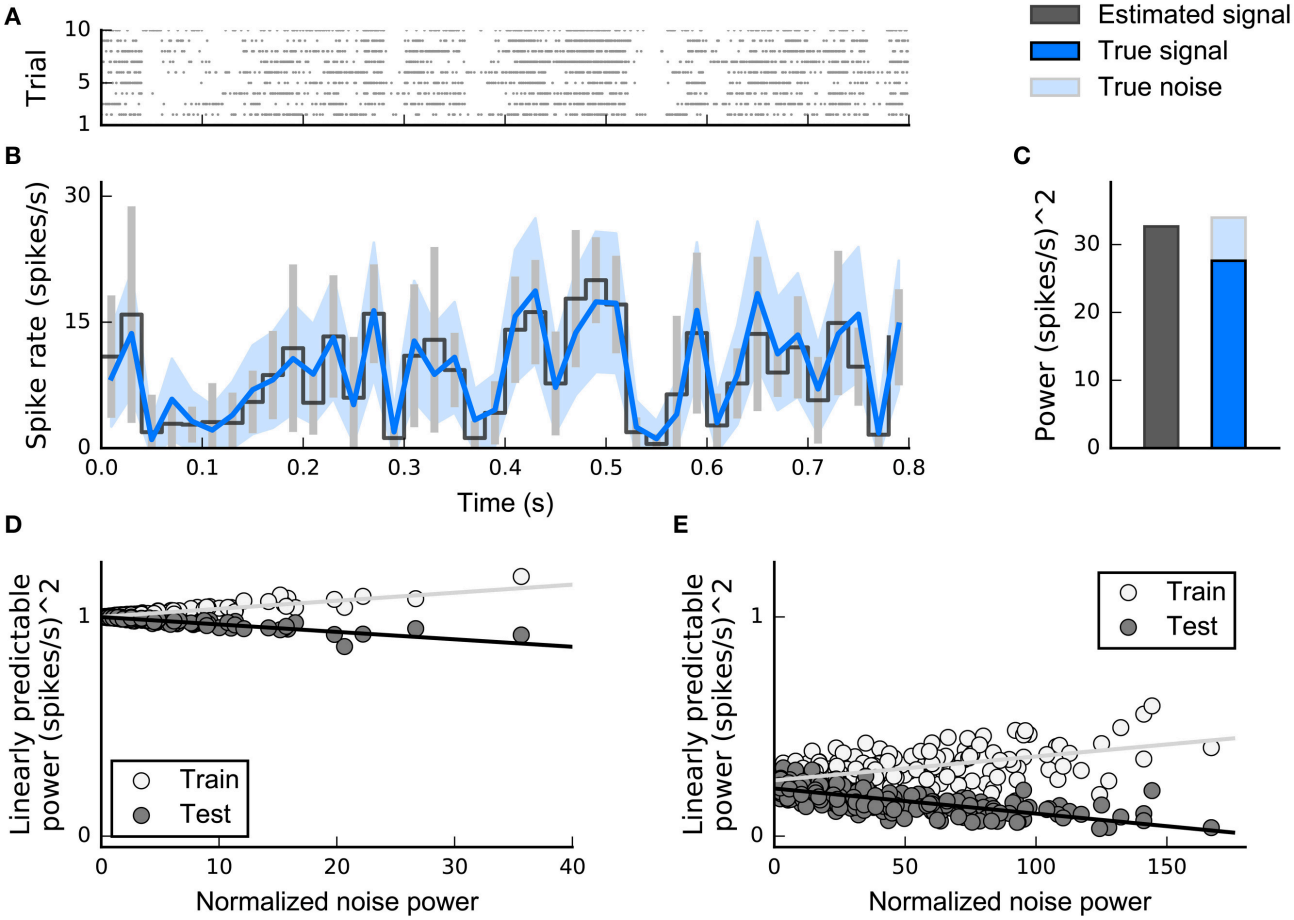
**Signal power, noise power, and population extrapolation**. Simulated data illustrate principle of quantification of predictable signal power. **(A)** Raster plot showing Poisson spike trains for 10 presentations of the same stimulus. **(B)** True signal (solid blue line) that was used to generate the spike trains, together with true noise (blue shaded area). The spike rate (black line) and the standard deviation across trials (gray bars) were estimated by counting spikes in discrete bins. **(C)** Power of estimated response, true signal, and true noise. The estimated response power overestimates the true signal power by the noise power (additivity of variances, see text). The estimated signal power is found by subtracting the noise power from the estimated response power. **(D)** Normalized predictive power for a population of 200 simulated linear-Gaussian cells. Predictions on training data (light gray circles) and testing data (dark gray circle) were done using a linear estimator. As expected, extrapolation to zero noise power reveals that the model accounts for the maximum linearly predictable power. **(E)** The same as in **(D)** but responses were simulated using 200 linear-nonlinear Bernoulli cells and a non-Gaussian stimulus (similar to the stimulus in Figure 3B). Extrapolation to the zero noise condition indicates that imperfect model performance is due to an incorrect model assumption (linear-Gaussian model) rather than to noise.

First, the simple property of additivity of variances implies that on each trial $\sigma_{r}^{2}\;\overset{E}{=}\sigma_{\mu }^{2}+\sigma_{\eta }^{2}$ where $\sigma_{\eta }^{2}$ is the average squared deviation from μ(*t*). Here $\sigma_{r}^{2}$ (as in Equation 26) is a noisy observed sum-of-squares depending on the particular response on a single trial, while $\sigma_{\mu }^{2}$ and $\sigma_{\eta }^{2}$ are expected measures of variance in the idealized response. Thus, $\overset{E}{=}$ means “equal in expectation”; the equality may not hold on any trial, but the expected values of the left- and right-hand sides are equal. This relationship depends only on the noise component having been defined to have zero expectation, and holds even if the variance or other property of the noise depends on the signal strength as would be expected for a Poisson noise process (see the simulated example in Figures 10A–C). We now construct two observed trial-averaged quantities, similar to the sum-of-squares terms used in the analysis of variance (ANOVA) (e.g., Lindgren, 1993): the power of the average response $\sigma_{\bar{r}}^{2}$, and the average power per response $\bar{\sigma_{r}^{2}}$, with $\bar{\cdot }$ indicating trial averages:

$$\sigma_{\bar{r}}^{2}\;\underline{\underline{\varepsilon }}\;\sigma_{\mu }^{2}+\sigma_{\bar{\eta }}^{2}\text{ and }\bar{\sigma_{r}^{2}}\;\underline{\underline{\varepsilon }}\;\sigma_{\mu }^{2}+\;\bar{\sigma_{\eta }^{2}}.$$

Assuming that the noise in each trial is independent, although the noise in different time bins within a trial need not be, we have: $\sigma_{\bar{\eta }}^{2}\;\overset{E}{=}\;\bar{\sigma_{\eta }^{2}}/N$. Then solving these two equations for $\sigma_{\mu }^{2}$ suggests the following estimator for the signal power:

(31) $$\hat{\sigma_{\mu }^{2}}=\frac{1}{N-1}\left(N\sigma_{\bar{r}}^{2}-\;\bar{\sigma_{r}^{2}}\right).$$

A similar estimator for the *noise power* is obtained by subtracting this expression from $\bar{\sigma_{r}^{2}}$. Thus, the resulting estimator of the fraction of explainable response power captured by a model, the *predictive power*, is given by

(32) $$\beta =\frac{\sigma_{r}^{2}-\sigma_{e}^{2}}{\hat{\sigma_{\mu }^{2}}}.$$

This corresponds to the *R*^2^ estimator (Equation 26) except that the explained variance is measured against an estimate of the stimulus-driven power (or variance) instead of the total response variance, which overestimates the signal power by the noise power (Figure 10C).

Hsu et al. (2004) applied a similar idea to the coherence measure (see Equation 28) to obtain an estimate of the coherence between model prediction and signal-driven response. However, it is important to note that whereas the estimator for the signal power itself (Equation 31) depends linearly on the measured power in single responses and their trial average and so is unbiased, estimators for the predictive power (Equation 32) and coherence (Hsu et al., 2004) which involve nonlinear transformation are at best consistent. However simulations (Figure 10D and Hsu et al., 2004) suggest that any finite-data biases might be small for typical data volumes.

David and Gallant (2005) study the bias in the correlation coefficient between (unregularized) prediction and validation measurements, using an analysis similar to the predictive power. They focus separately on the prediction errors introduced directly by noise in the measured validation data and by mis-estimation of model parameters from noisy training data, and propose two different schemes for extrapolation in number of trials or training time (though not in the population noise level as described below). While they arrive at the correct estimate of the correlation coefficient of the ideal model, this approach makes assumptions that might not hold for many experimental data sets. First, the unregularized model is assumed to be predictive which is often not the case for realistic data sizes (see Regularization). Second, the (linear) model fit is assumed to be the same in the noise-free training and validation sets. This is approximately true for large training and validation data sets, but unlikely for rather limited amounts of data as stimuli in the two sets might differ substantially and neural models are stimulus dependent (Christianson et al., 2008).

#### Upper and lower predictive power estimates

Model parameters (such as the weights or coefficients of the SRF) are commonly estimated by minimizing the mean squared error of the model prediction on the training data. By definition, these least-mean-squares (LMS) parameters produce model predictions for the training data that have minimum possible error, and therefore maximal predictive power. Of course, the resulting maximal value, the training predictive power, will inevitably include an element of overfitting to the training data, and so will overestimate the true predictive power of the model with ideal parameters (i.e., the model that would perform best on average for all possible stimulus-response combinations, not just the training data). More precisely, the expected value of the training predictive power of the LMS parameters is an upper bound on the predictive power of the model with ideal parameters. Thus, the measured training predictive power can be considered an upper estimate of the true predictive power of the model class (light gray dots in Figures 10D,E).

We can also obtain a lower estimate, defined similarly, by empirically measuring the generalization performance of the model by cross-validation. Cross-validation provides an unbiased estimate of the average generalization performance of the fitted models (as obtained from the training fraction of the available data). Since these models are inevitably overfit to their training data, not the test data, the expected value of this cross-validation predictive power bounds the predictive power of the model with ideal parameters from below, and thereby provides the desired lower estimate of the true predictive power of the model class (dark gray dots in Figures 10D,E).

#### Population extrapolation

For any one recording of finite length, the true predictive power of the model class (i.e., the predictive power of the version of the model with ideal parameters) can only be bracketed between the upper and lower estimates defined above. The looseness of these estimates will depend on the variability or noise in the recording. For a recording with high trial-to-trial variability, the model parameters will be more strongly overfit to the noise in the training data. Thus, we expect the training predictive power on such a recording to appear high relative to the signal power, and the cross-validation predictive power to appear low. Indeed, in very high-noise conditions, the model may primarily describe the stimulus-independent noisy part of the training data, and so the training predictive power might exceed the estimated signal power, while the cross-validation predictive power may fall below zero (that is, the predictions made by the model may be worse than a simple unchanging mean rate prediction). Thus, the estimates may not usefully constrain the predictive power measure for a particular recording.

However, the systematic dependence of the bounds on the variability of the neural response makes it possible to tighten the estimates of model predictive power for the population as a whole. Rather than simply averaging the bounds — and thus the effects of noise—across neurons, the upper and lower estimates of model predictive power are regressed as a function of noise level and extrapolated to the zero-noise intercept. This extrapolation yields a relatively tight estimated range within which the optimal population mean predictive performance of the model must lie, while discounting the influence of variability on the assessed performance. This extrapolation is illustrated for linear models fit to two simulated populations in Figures 10D,E. The second of these populations was designed to be nonlinear, and this is reflected in the low values of extrapolated linear predictive power. In practice, the extrapolated upper and lower bounds may not always converge to the same value: if the true response function is very far from the hypothesized model class, then a model fit to finite data, even if noiseless, would generalize to novel stimuli more poorly than it fits the training data.

#### Model mismatch and RF estimates

We have emphasized previously that a tractable model class can be expected at best only to approximate the true SRF of a neuron. To what extent, then, do RF estimates in such models still yield useful qualitative indications of the true neural response properties?

**Figure 11:**
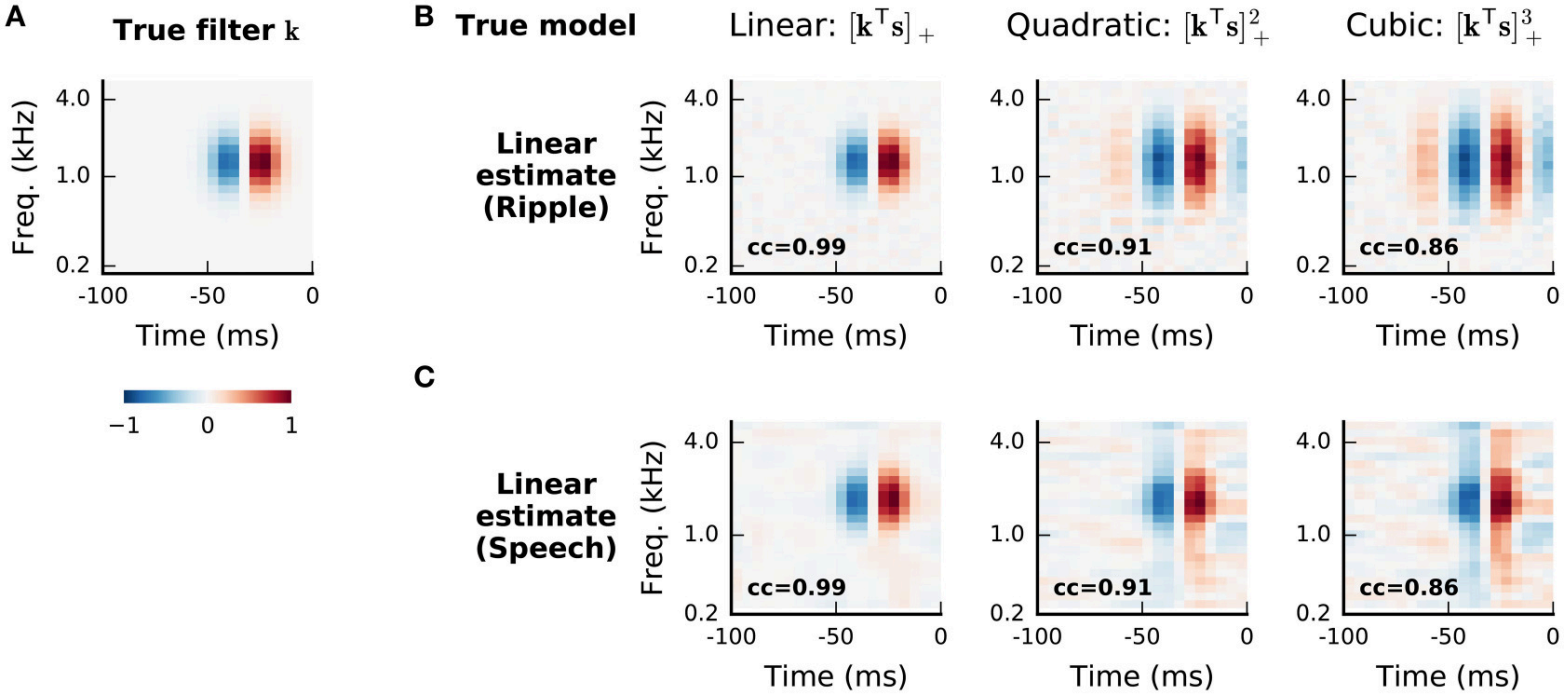
**Simulations illustrating the effect of model mismatch on RF estimates**. **(A)** True spectro-temporal receptive field used in the simulations. **(B)** Estimates obtained using a linear estimator for a threshold linear, a threshold-quadratic, and a threshold cubic-Poission model with rate estimated from 20 trials (*p*(spike) = 0.1). Stimuli were dynamic ripples, a sound class modulated sinusoidally in both the temporal and spectral domains. [·]_+_ denotes half-wave rectification. **(C)** The same as in **(B)** but for human speech stimuli. For both stimulus classes a model mismatch can result in stimulus-dependent overestimation of the extent of the true RF. The cc values stated are the Pearson correlation coefficients between true and estimated receptive fields.

In general, the RF estimate for a mismatched model depends both on the true neuronal SRF and on the stimulus ensemble (Christianson et al., 2008). This is most evident for a linear model, as the slope of the best linear approximation to a nonlinear curve must clearly depend on the domain over which the approximation is made. In the high-dimensional setting, estimated weights depend not only on the range and distribution of stimulus values along each dimension (e.g., Figure 3) but potentially also on statistical interactions between dimensions. Thus, estimates of weights along stimulus dimensions which fall *outside* the true RF (and so do not actually affect firing at all) may be non-zero if the stimulus along those dimensions correlates with a relevant nonlinear function of the stimulus *within* the true RF. This phenomenon can lead to striking artifacts even in the “autocorrelation corrected” linear estimate (Figure 11).

The same effect applies to nonlinear models, even when the estimator is consistent or unbiased (as these concepts are meaningful only for properly matched models). For example, the MID (or the equivalent LNP MLE) will generally depend on stimulus statistics if the true neuronal SRF is not LNP. It follows that differences in estimated RFs measured under different stimulus conditions, even using a method such as MID, may not necessarily reflect adaptive changes in the true SRF (Borst et al., 2005; Christianson et al., 2008). To identify genuine SRF changes, models must be fit using the same distribution of stimuli placed within different adaptive contexts.

We observed above that the MLE for a linear model also provides an unbiased estimate of the RF filter of an LNP model when the stimulus ensemble is elliptically symmetric. Might a similar principle apply more generally? Indeed, stimuli that are chosen independently along each input dimension do provide a (somewhat weaker) guarantee. Provided that the SRF model estimator allows for separate adjustment of its RF components along each stimulus dimension (and is not, for example, subject to a regularization constraint in which RF weights are assumed to vary smoothly), the RF estimated using such a stimulus will not systematically overestimate dependence on stimulus dimensions outside the true RF (Christianson et al., 2008). However, RF weights estimated within the true responsive region will not necessarily reflect the true quantitative influence of the stimulus dimensions. As Christianson et al. (2008) point out, except in the special case of Gaussian stimuli, dimensional independence depends not only on the stimulus ensemble but also on the choice of dimensions. Thus, the ripple stimuli employed in Figure 11B are not independent in the spectrotemporal domain, but are close to being so (and indeed, a random sparse combination of ripples would be exactly so) in the spectrotemporal modulation domain: the Fourier transform space of the spectrogram. It follows that the use of ripple stimuli will not lead to an overestimate of the extent of the modulation RF.

## Discussion

Abstract stimulus–response function models can be versatile and powerful tools for addressing many different questions about sensory processing and neural representation. The great advantage of these models is that their parameters can be estimated from experimentally feasible amounts of data, but nevertheless can describe neuronal responses across a large subset of a high-dimensional stimulus space. The disadvantage is the obverse of this advantage; the same abstract formulation that permits robust and efficient parameter estimation from limited data also requires assumptions that can produce potentially misleading results arising from mismatch with biological reality.

Unlike biophysical models that describe actual low-level mechanisms of sensory processing such as synaptic transmission and channel dynamics, functional models are abstract descriptors of the stimulus–response function transformation. In general, then, the estimated parameters of functional models should not be interpreted as estimates of specific physical properties of the biological system. The true test of a stimulus–response function model is not whether the fitted parameters can be mapped onto low-level biological mechanisms, but whether the model can successfully predict neuronal responses to novel instances of the sensory input. This review has included a summary of means by which the quality of model predictions can be rigorously and systematically quantified, in a manner robust to the level of stimulus-independent “noise” in the neuronal responses. Such methods for evaluating model predictive power—combined with a healthy appreciation for the potential issues arising from model mismatch—help to make abstract stimulus–response function models an essential tool in the arsenal of methods for analysis of neural systems.

### Data sharing

Software implementing many of the estimators described above is available online at http://www.gatsby.ucl.ac.uk/resources/srf/.

## Author contributions

AFM and MS implemented the estimation methods. AFM implemented and conducted all simulations and analyses and created all figures in the manuscript. All four authors wrote the manuscript.

## Funding

This work was supported by Gatsby Charitable Foundation (MS), Simons Foundation (SCGB323228; MS), Action on Hearing Loss (G77, F44, F61; JFL), and National Institutes of Health (F32-DC015376; RSW).

### Conflict of interest statement

The authors declare that the research was conducted in the absence of any commercial or financial relationships that could be construed as a potential conflict of interest.
